# Adipose tissue distribution from body MRI is associated with cross-sectional and longitudinal brain age in adults

**DOI:** 10.1016/j.nicl.2022.102949

**Published:** 2022-01-27

**Authors:** Dani Beck, Ann-Marie G. de Lange, Dag Alnæs, Ivan I. Maximov, Mads L. Pedersen, Olof Dahlqvist Leinhard, Jennifer Linge, Rozalyn Simon, Geneviève Richard, Kristine M. Ulrichsen, Erlend S. Dørum, Knut K. Kolskår, Anne-Marthe Sanders, Adriano Winterton, Tiril P. Gurholt, Tobias Kaufmann, Nils Eiel Steen, Jan Egil Nordvik, Ole A. Andreassen, Lars T. Westlye

**Affiliations:** a NORMENT, Division of Mental Health and Addiction, Oslo University Hospital & Institute of Clinical Medicine, University of Oslo, Norway; b Department of Psychology, University of Oslo, Norway; c Sunnaas Rehabilitation Hospital HT, Nesodden, Norway; d LREN, Centre for Research in Neurosciences-Department of Clinical Neurosciences, CHUV and University of Lausanne, Lausanne, Switzerland; e Department of Psychiatry, University of Oxford, Warneford Hospital, Oxford, UK; f Bjørknes College, Oslo, Norway; g Department of Health and Functioning, Western Norway University of Applied Sciences, Bergen, Norway; h AMRA Medical AB, Linköping, Sweden; i Center for Medical Image Science and Visualization (CMIV), Linköping University, Linköping, Sweden; j Department of Health, Medicine, and Caring Sciences, Linköping University, Linköping, Sweden; k Department of Psychiatry and Psychotherapy, University of Tübingen, Germany; l CatoSenteret Rehabilitation Center, Son, Norway; m KG Jebsen Centre for Neurodevelopmental Disorders, University of Oslo, Norway

**Keywords:** Adipose tissue, Obesity, T1, DTI, MRI, Brain age

## Abstract

There is an intimate body-brain connection in ageing, and obesity is a key risk factor for poor cardiometabolic health and neurodegenerative conditions. Although research has demonstrated deleterious effects of obesity on brain structure and function, the majority of studies have used conventional measures such as waist-to-hip ratio, waist circumference, and body mass index. While sensitive to gross features of body composition, such global anthropometric features fail to describe regional differences in body fat distribution and composition. The sample consisted of baseline brain magnetic resonance imaging (MRI) acquired from 790 healthy participants aged 18–94 years (mean ± standard deviation (SD) at baseline: 46.8 ± 16.3), and follow-up brain MRI collected from 272 of those individuals (two time-points with 19.7 months interval, on average (min = 9.8, max = 35.6). Of the 790 included participants, cross-sectional body MRI data was available from a subgroup of 286 participants, with age range 19–86 (mean = 57.6, SD = 15.6). Adopting a mixed cross-sectional and longitudinal design, we investigated cross-sectional body magnetic resonance imaging measures of adipose tissue distribution in relation to longitudinal brain structure using MRI-based morphometry (T1) and diffusion tensor imaging (DTI). We estimated tissue-specific brain age at two time points and performed Bayesian multilevel modelling to investigate the associations between adipose measures at follow-up and brain age gap (BAG) – the difference between actual age and the prediction of the brain’s biological age – at baseline and follow-up. We also tested for interactions between BAG and both time and age on each adipose measure. The results showed credible associations between T1-based BAG and liver fat, muscle fat infiltration (MFI), and weight-to-muscle ratio (WMR), indicating older-appearing brains in people with higher measures of adipose tissue. Longitudinal evidence supported interaction effects between time and MFI and WMR on T1-based BAG, indicating accelerated ageing over the course of the study period in people with higher measures of adipose tissue. The results show that specific measures of fat distribution are associated with brain ageing and that different compartments of adipose tissue may be differentially linked with increased brain ageing, with potential to identify key processes involved in age-related transdiagnostic disease processes.

## Highlights

• There is an intimate body-brain connection in ageing, and obesity is a key risk factor for poor cardiometabolic health and neurodegenerative conditions.
• We investigated adipose tissue distribution from body magnetic resonance imaging (MRI) in relation to brain structure using MRI-based morphometry and diffusion tensor imaging (DTI).
• The results indicated older-appearing brains in people with higher measures of adipose tissue, and accelerated ageing over the course of the study period in people with higher measures of adipose tissue.

## 1 Introduction

An increasing body of evidence supports an intimate body-brain connection in ageing, with cardiovascular disease (CVD), cognitive decline, and dementia sharing various cardiometabolic risk factors (Qiu & Fratiglioni, 2015). Among these, obesity subsists as a key risk factor (Bhupathiraju and Hu, 2016, Luppino et al., 2010), with evidence extending the links to include mental disorders (Bahrami et al., 2020, Ditmars et al., 2021, Luppino et al., 2010, Perry et al., 2021, Quintana et al., 2017, Rajan and Menon, 2017, Ringen et al., 2018, Scott et al., 2008) and age-related neurocognitive and neurological conditions including dementia and stroke (Anstey et al., 2011, Strazzullo et al., 2010).

Higher adipose tissue levels as indexed by waist circumference (WC), waist-to-hip ratio (WHR), body mass index (BMI), and increased abdominal subcutaneous adipose tissue (ASAT) and visceral adipose tissue (VAT) measures have all been associated with global brain volume decreases (Debette and Markus, 2010, Gunstad et al., 2005, Mulugeta et al., 2021, Ward et al., 2005). Moreover, regional findings have consistently shown negative associations between obesity and brain grey matter volume (Gurholt et al., 2020, Pannacciulli et al., 2006, Taki et al., 2008, Walther et al., 2010), white matter microstructure, including reduced white matter tract coherence (Friedman et al., 2014, Willette and Kapogiannis, 2015), white matter integrity (Marks et al., 2011, Stanek et al., 2011, Xu et al., 2013), microstructural changes in childhood (Rapuano et al., 2020), and increased axonal and myelin damage (Mueller et al., 2011, Xu et al., 2013) based on diffusion MRI. White matter volumetric studies have revealed less consistent findings, reporting positive (Walther et al., 2010), negative (Raji et al., 2010), as well as no (Gunstad et al., 2005) significant associations between brain white matter volume and adiposity.

While there is a wealth of research focusing on conventional anthropometric measures such as BMI, not all individuals with a higher BMI have the same disease risk (Mulugeta et al., 2021). A study assessing 27,000 individuals from 52 countries identified abdominal obesity as one of nine key risk factors that accounted for most of the risk of myocardial infarction worldwide (Yusuf et al., 2004). However, while some obese individuals develop health problems such as lipid abnormalities and type 2 diabetes (Iacobini et al., 2019), others are metabolically healthy. Partly motivated by this heterogeneity and complexity of fat distribution and cardiometabolic health, body MRI has recently emerged as a novel opportunity to investigate adipose tissue distribution beyond anthropometric measures (Linge et al., 2018, Linge et al., 2021, Linge et al., 2020, Linge et al., 2019).

Research utilising body MRI has found associations between VAT and muscle fat infiltration (MFI) and coronary heart disease (CHD) and type 2 diabetes (T2D) (Linge et al., 2018). Moreover, higher liver fat has been associated with T2D and lower liver fat with CHD (Linge et al., 2018). Cross-sectional analyses investigating body and brain MRI associations have reported negative associations between liver fat, MFI and cerebral cortical thickness while thigh muscle volume (TMV) was positively associated with brain stem and accumbens volumes (Gurholt et al., 2020).

Advanced multimodal brain MRI provides a wealth of information reflecting structural and functional characteristics of the brain. Brain age prediction using machine learning and a combination of MRI features provides a reliable approach for reducing the complexity and dimensionality of imaging data. The difference between the brain-predicted age and an individual’s chronological age, also referred to as the brain age gap (BAG), can be used to assess deviations from expected age trajectories, with potential utility in studies of brain disorders and ageing (Cole et al., 2017, Kaufmann et al., 2019). This has clear clinical implications for patient groups, where studies have reported larger brain age gaps in patients with various neurological and psychiatric disorders (Han et al., 2021, Høgestøl et al., 2019, Kaufmann et al., 2019, Pardoe et al., 2017, Sone et al., 2021, Tønnesen et al., 2020).

While brain age research has frequently merged individual and age-related differences using cross-sectional data, recent evidence has demonstrated that the rate of brain ageing may be dependent on a range of life events and lifestyle factors (Cole, 2020, Sanders et al., 2021), and characteristics related to cardiovascular health and obesity, including WHR and BMI (Beck et al., 2021a, de Lange et al., 2020, Franke et al., 2014, Kolbeinsson et al., 2020, Kolenic et al., 2018, Ronan et al., 2016).

The motivation of the current study was to utilise more effective and precise descriptions of fat distribution in order to localise the subtypes of body composition that impact brain health and ageing. Our primary aim was to identify interactions between adipose tissue measures based on body MRI and tissue specific (DTI and T1-weighted) measures of brain age. We investigated cross-sectional associations of tissue specific BAG and detailed adipose tissue measures (body composition) and, for comparison, conventional anthropometric measures (BMI and WHR) used in a recent study (Beck et al., 2021a). Next, we tested for associations between longitudinal brain age and body composition at follow-up and investigated associations between each adiposity measure and longitudinal BAG. Adopting a Bayesian statistical framework, we hypothesised that higher fat ratio, total abdominal adipose volume index (total adipose), weight-to-muscle ratio (WMR), VAT index, MFI, and liver fat percentage (liver fat) would be associated with older appearing brains, with stronger associations in the body MRI measures than for traditional anthropometric features. Further, we hypothesised that higher fat ratio, total adipose, WMR, VAT index, MFI, and liver fat are associated with accelerated brain ageing as reflected by larger changes in BAG between baseline and follow-up.

## 2 Material and methods

### 2.1 Sample description

Two integrated studies – the Thematically Organised Psychosis (TOP) (Tønnesen et al., 2018) and StrokeMRI (Richard et al., 2018) – formed the initial sample, which included 1130 brain MRI datasets from 832 healthy participants. All procedures were conducted in line with the Declaration of Helsinki and the study has been approved by the Norwegian Regional Committee for Medical and Health Research Ethics (REC). All participants provided written informed consent, and exclusion criteria included neurological and mental disorders, and previous head trauma.

**Fig. 1:**
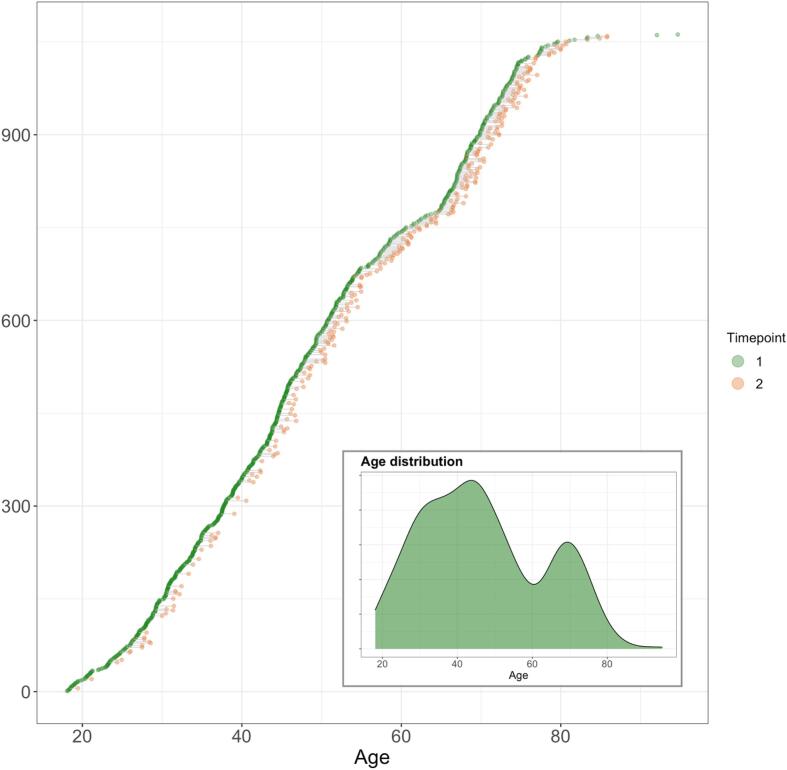
Available baseline and follow-up data. All participants are shown. Participants with data at baseline are visualised in green dots (N = 790). Of these participants, those with longitudinal measures of brain MRI are connected to corresponding timepoint two orange dots (N = 272). The y axis shows index which reorders data to sort by age at first timepoint. Subplot shows density of age distribution at baseline. (For interpretation of the references to colour in this figure legend, the reader is referred to the web version of this article.)

Following the removal of 68 datasets after quality checking (QC) of the MRI data (see section 2.5), the final sample included 1062 brain MRI datasets collected from 790 healthy participants aged 18–94 years (mean ± standard deviation (SD) at baseline: 46.8 ± 16.3). This included longitudinal data (two time-points with 19.7 months interval, on average (min = 9.8, max = 35.6) from 272 participants. Of the 790 included participants, body MRI data was available from a subgroup of 286 participants, with age range 19–86 (mean = 57.6, SD = 15.6). Demographic information is summarised in Table 1 and Fig. 1.

**Table 1:** Descriptive characteristics of the study sample.

	Baseline brain MRI	Follow-up brain MRI	Body MRI	Males at baseline	Females at baseline	Males at follow-up	Females at follow-up
*N* subjects	790	272	286	372 (47.1%)	418 (52.9%)	106 (39%)	166 (61%)
Sex (Males/Females)	372/418	106/166	110/176	372/0	0/418	106/0	0/166
Age (mean ± SD)	46.7 ± 16.3	56.9 ± 15.0	57.6 ± 15.6	45.4 ± 16.3	48.0 ± 16.3	56.7 ± 16.9	57.1 ± 13.6
Predicted Age T1	46.6 ± 17.4	56.7 ± 16.8	57.8 ± 17.7	44.6 ± 16.8	48.4 ± 17.3	55.3 ± 17.9	57.6 ± 16.0
Predicted Age DTI	46.5 ± 16.9	57.0 ± 15.9	58.2 ± 16.9	46.9 ± 16.7	47.1 ± 17.0	57.7 ± 17.3	56.6 ± 15.0
BAG T1	−0.15 ± 6.4	−0.23 ± 6.9	0.2 ± 6.9	−0.8 ± 5.9	0.5 ± 6.9	−1.41 ± 6.4	0.53 ± 7.2
BAG DTI	−0.2 ± 5.1	0.05 ± 0.9	0.5 ± 5.4	0–5 ± 5.1	−0.9 ± 5.0	0.92 ± 5.0	−0.51 ± 5.4
BMI, kg/m^2^	25.2 ± 4.06	25.0 ± 3.7		25.6 ± 3.7	24.8 ± 4.3	25.3 ± 2.9	25.3 ± 4.4
Waist-to-hip ratio (WHR)	0.87 ± 0.09	0.9 ± 0.09		0.91 ± 0.1	0.83 ± 0.1	0.93 ± 0.07	0.86 ± 0.08
Visceral adipose tissue (VAT) (min–max)			2.7 (0.4–9.2)	3.7 (0.6–9.2)	2.2 (0.4–5.5)		
Abdominal subcutaneous adipose tissue (ASAT)			6.3 (1.1–19.7)	4.9 (1.5–13.4)	7.1 (1.1–19.8)		
Thigh muscle volume (TMV)			2.6 (0.6–1.5)	3.3 (2.3–4.2)	2.2 (1.5–3.3)		
Weight-to-muscle ratio (WMR)			29.3 (12.0–82.5)	25.6 (19.4–33.9)	31.7 (12.0–82.5)		
Liver PDFF (Liver fat)			4.3 (1.1–29.8)	4.9 (1.1–29.8)	3.9 (1.24–27.8)		
Fat ratio			74.5 (34.9–90.5)	70.0 (34.9–84.7)	77.5 (42.7–90.5)		
Visceral abdominal adipose tissue index (VAT index)			0.9 (0.14–2.9)	1.1 (0.2–2.9)	0.8 (0.14–2.0)		
Total abdominal adipose tissue index (Total adipose)			3.0 (0.51–8.5)	2.6 (0.57–5.3)	3.3 (0.51–8.5)		
Muscle fat infiltration (MFI)			7.6 (3.6–17.2)	6.9 (3.6–12.8)	8.3 (4.5–17.2)

An independent training sample from the Cambridge Centre for Ageing and Neuroscience (Cam-CAN: http://www.mrc-cbu.cam.ac.uk/datasets/camcan/; Shafto et al., 2014, Taylor et al., 2017) was used for brain age prediction (section 2.6). After QC, MRI data from 622 participants were included (age range = 18–87, mean age ± standard deviation = 54.2 ± 18.4).

### 2.2 MRI acquisition

MRI was performed at Oslo University Hospital, Norway, on a GE Discovery MR750 3T scanner. Brain MRI was collected with a 32-channel head coil. T1-weighted data were acquired with a 32-channel head coil using a 3D inversion recovery prepared fast spoiled gradient recalled sequence (IR-FSPGR; BRAVO) with the following parameters: TR: 8.16 ms, TE: 3.18 ms, flip angle: 12°, voxel size: 1 × 1 × 1 mm^3^, FOV: 256 × 256 mm^2^, 188 sagittal slices, scan time: 4:43 min. DTI data were acquired with a spin echo planar imaging (EPI) sequence with the following parameters: repetition time (TR)/echo time (TE)/flip angle: 150 ms/83.1 ms/90°, FOV: 256 × 256 mm^2^, slice thickness: 2 mm, in-plane resolution: 2 × 2 mm, 60 non-coplanar directions (b = 1000 s/mm^2^) and 5b = 0 volumes, scan time: 8:58 min. In addition, 7b = 0 volumes with reversed phase-encoding direction were acquired.

Body MRI was performed on a GE Discovery MR750 3 T scanner. A 3D dual-echo LAVA Flex pulse sequence was carried out to acquire water and fat separated volumetric data covering neck to knees acquired in X stacks using breath hold acquisition in the abdominal region. The following parameters were used: TE: Min Full, flip angle: 10°, FOV: 50 × 50 mm, slice thickness: 5 mm, total scan time: approx. 6:00 min. For proton density fat fraction (PDFF) assessment in the liver, a single-slice 3D multi-echo IDEAL IQ pulse sequence was used with the following parameters: TE: Minimum, flip angle: 3°, FOV: 45 × 45 mm^2^, slice thickness: 8 mm, scan time: 0:22 min.

The Cam-CAN training set participants were scanned on a 3 T Siemens TIM Trio scanner with a 32- channel head-coil at Medical Research Council (UK) Cognition and Brain Sciences Unit (MRC-CBSU) in Cambridge, UK. High-resolution 3D T1-weighted data was collected using a magnetisation prepared rapid gradient echo (MPRAGE) sequence with the following parameters: TR: 2250 ms, TE: 2.99 ms, inversion time (TI): 900 ms, flip angle: 9°, FOV of 256 × 240 × 192 mm; voxel size = 1 × 1 × 1 mm, GRAPPA acceleration factor of 2, scan time 4:32 min (Dixon et al., 2014). DTI data was acquired using a twice-refocused spin echo sequence with the following parameters: TR: 9100 ms, TE: 104 ms, FOV: 192 × 192 mm, voxel size: 2 mm, 66 axial slices using 30 directions with b = 1000 s/mm^2^, 30 directions with b = 2000 s/mm^2^, and 3b = 0 images (Dixon et al., 2014).

### 2.3 DTI processing and TBSS analysis

Processing steps for single-shell diffusion MRI data in the test set followed a previously described pipeline (Maximov et al., 2019), including noise correction (Veraart et al., 2016), Gibbs ringing correction (Kellner et al., 2016), corrections for susceptibility induced distortions, head movements and eddy current induced distortions using topup (http://fsl.fmrib.ox.ac.uk/fsl/fslwiki/topup) and eddy (http://fsl.fmrib.ox.ac.uk/fsl/fslwiki/eddy) (Andersson & Sotiropoulos, 2016). Isotropic smoothing was carried out with a Gaussian kernel of 1 mm^3^ implemented in the FSL function *fslmaths.* DTI metrics were estimated using *dtifit* in FSL and a weighted least squares algorithm. Processing steps for the training set followed a similar pipeline with the exception of the noise correction procedure.

Voxelwise analysis of the fractional anisotropy (FA) data was carried out using Tract-Based Spatial Statistics (TBSS) (Smith et al., 2006), as part of FSL (Smith et al., 2004). First, FA images were brain-extracted using BET (Smith, 2002) and aligned into a common space (FMRI58_FA template) using the nonlinear registration tool FNIRT (Andersson, Jenkinson, & Smith., 2007; Jenkinson et al., 2012), which uses a b-spline representation of the registration warp field (Rueckert et al., 1999). Next, the mean FA volume of all subjects was created and thinned to create a mean FA skeleton that represents the centres of all tracts common to the group. Each subject's aligned FA data was then projected onto this skeleton. The mean FA skeleton was thresholded at FA > 0.2. This procedure was repeated in order to extract axial diffusivity (AD), mean diffusivity (MD), and radial diffusivity (RD). *fslmeants* was used to extract the mean skeleton and 20 regions of interest (ROI) based on a probabilistic white matter atlas (JHU) (Hua et al., 2008) for each metric. Including the mean skeleton values, 276 features (see Supplementary material; SI Table 1) per individual were derived in total.

### 2.4 FreeSurfer processing

T1-weighted MRI data were processed using FreeSurfer (Fischl, 2012) version 7.1.0 for the test set and version FreeSurfer version 5.3 for the training set. To extract reliable area, volume and thickness estimates, the test set including follow-up data were processed with the longitudinal stream (Reuter et al., 2012) in FreeSurfer. Specifically, an unbiased within-subject template space and image (Reuter & Fischl, 2011) is created using robust, inverse consistent registration (Reuter et al., 2010). Several processing steps, such as skull stripping, Talairach transforms, atlas registration as well as spherical surface maps and parcellations are then initialised with common information from the within-subject template, significantly increasing reliability and statistical power (Reuter et al., 2012). Due to the longitudinal stream in FreeSurfer influencing the thickness estimates, and subsequently having an impact on brain age prediction (Høgestøl et al., 2019), both cross-sectional and longitudinal data in the test set were processed with the longitudinal stream. All reconstructions were visually assessed and edited by trained research personnel. Cortical parcellation was performed using the Desikan-Killiany atlas (Desikan et al., 2006), and subcortical segmentation was performed using a probabilistic atlas (Fischl et al., 2002). In total, 269 FreeSurfer (SI Table 1) based features were extracted, including global features for intracranial volume, total surface area, and whole cortex mean thickness, as well as the volume of subcortical structures.

### 2.5 Quality control (QC) procedure

A detailed description of the complete QC procedure for the final sample is available in (Beck et al., 2021a). Briefly, for DTI we derived various QC metrics (SI Table 2), including temporal signal-to-noise-ratio (tSNR) (Roalf et al., 2016) to flag data deemed to have unsatisfactory quality. For T1-weighted data, QC was carried out using the ENIGMA cortical QC protocol including three major steps: outlier detection, internal surface method, and external surface method. Following the removal of datasets with inadequate quality (n = 30), the separate T1 and DTI datasets were merged with BMI and WHR measures, leaving the final sample used for the study at N = 1062 datasets from 790 individuals, among which N = 286 had body MRI data available.

Body MRI QC was carried out using a multivariate outlier detection algorithm, where anomalies in the data are detected as observations that do not conform to an expected pattern to other items. Using the R package *mvoutlier* (Filzmoser et al., 2005), potential outliers were flagged using the Mahalanobis distance (SI Figs. 1 and 2). Informed by an interactive plot using the *chisq.plot* function, manual outlier observations of each of these flagged values deemed none of them as true outliers, leading to no further removal from the initial 286 body MRI dataset.

### 2.6 Brain age prediction

We performed brain age prediction using T1-weighted and DTI data using XGBoost regression (https://xgboost.readthedocs.io/en/latest/python), which is based on a decision-tree ensemble algorithm used in several recent brain age prediction studies (Beck et al., 2021a, de Lange et al., 2019, de Lange et al., 2020, Kaufmann et al., 2019, Richard et al., 2020). BAG was calculated using (predicted age – chronological age) for each of the models, providing T1 and DTI-based BAG values for each individual. The BAG estimates were residualised for age to account for age-bias (de Lange and Cole, 2020, Liang et al., 2019), where brain age is overestimated in younger subjects while they are underestimated in older subjects.

### 2.7 Adipose tissue measures

The raw data from 286 body MRI scans included the following measures: height, weight, left and right anterior thigh muscle volume, left and right anterior thigh muscle fat infiltration, left and right posterior thigh muscle volume, left and right posterior thigh muscle fat infiltration, visceral abdominal tissue adipose volume, abdominal subcutaneous adipose tissue volume, and liver fat fraction. Prior to being converted to the body composition features used for the study, the raw data was first inspected for missing entries. A missing data report (SI Fig. 3) revealed that no variable had more than 3.5% missing values. Next, missing values were imputed using the *MICE* package (van Buuren and Groothuis-Oudshoorn, 2011) in R, where five imputations were carried out using the predictive mean matching method (package default). The distribution of the original and imputed data was inspected (SI Fig. 4) and the imputed data were deemed as plausible values. Of the five imputations, the first was used for the remainder of the study.

To investigate the associations between the adipose tissue measures, hierarchical clustering of the raw variables bar height and weight was performed using *hclust*, part of the *stats* package in R (R Core Team, 2012), which uses the complete linkage method to form clusters. Five cluster groups were revealed (SI Fig. 5), including left and right anterior and posterior thigh muscle volumes, left and right anterior and posterior thigh muscle fat infiltration, liver fat, and visceral and subcutaneous abdominal tissue volume.

**Fig. 2:**
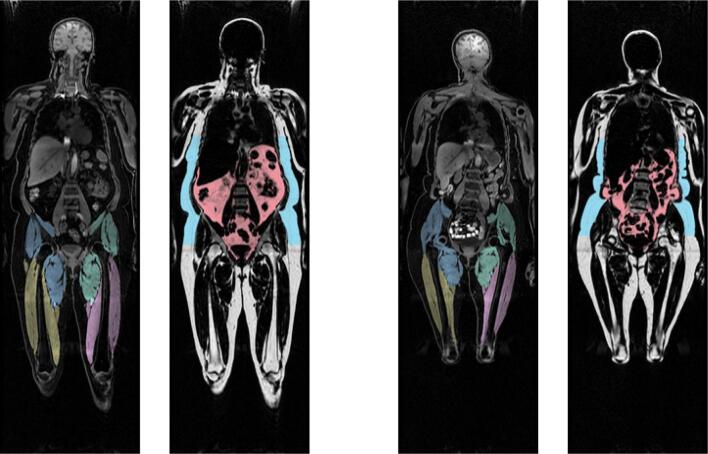
Body MRI. Showing two participants with a coronal slice from their MRI scan with VAT (pink) and ASAT (blue), and thigh muscle segmentations. (For interpretation of the references to colour in this figure legend, the reader is referred to the web version of this article.)

Informed by the cluster formations, left and right anterior and posterior thigh muscle and fat infiltration volumes were combined to form two average measures of thigh muscle volume and thigh muscle fat infiltration measures. Moreover, raw data was converted to body composition features following calculations provided in (Linge et al., 2018). The final adipose tissue measures included liver fat, describing the PDFF in the liver; visceral adipose tissue index (VAT index), which is VAT normalised by height^2^, describing the intra-abdominal fat surrounding the organs; total adipose, which is the total abdominal fat (VAT and ASAT) normalised by height^2^; weight-to-muscle ratio (WMR), which is body weight divided by thigh muscle volume; fat ratio, which is the total abdominal fat divided by total abdominal fat and thigh muscle volume; and muscle fat infiltration (MFI), representing intramuscular fat. Fig. 2 shows the body MRI for two participants. Fig. 5 shows the associations between the adiposity measures in a network correlation graph, created using the *qgraph* (Epskamp et al., 2012) R package. For a correlation matrix of adiposity measures see SI Fig. 6.

### 2.8 Statistical analysis

All statistical analyses were performed using R, version 3.6.0 (www.r-project.org/) (R Core Team, 2012). Bayesian multilevel models were carried out in *Stan* (Stan Development Team, 2019) using the *brms* (Bürkner, 2017, Bürkner, 2018) package, where multivariate models are fitted in familiar syntax to comparable frequentist approaches such as a linear mixed effects model using the *lme4* (Bates et al., 2015). A general overview of the brms package can be found in Bürkner (2017), while information pertaining to multilevel formula syntax implemented in the analysis can be found in Bürkner (2018).

To address the primary aim of the study, we tested for associations between tissue specific BAG and each adiposity measure. BAG (for T1 and DTI separately) was entered as the dependent variable with each adiposity measure separately entered as the independent fixed effects variable along with age, sex, and time, and subject ID as random effect. This was employed as following:

(1) $$\mathrm{BAG}\;Adipositymeasure+Age+Sex+TP\left(1\right|\mathrm{SubjectID})$$

To test our hypothesis that adiposity influences brain ageing we tested for associations between longitudinal changes in BAG and cross-sectional body MRI measures collected at follow-up using Bayesian multilevel models. Similarly, we tested for interactions between age and adiposity measures on BAG. For each of the models, BAG was entered as the dependent variable, with the first model running through interaction effect of each adiposity measure and time, and the second model running through interaction effect of each adiposity measure and age. Timepoint and age were controlled for in the models where appropriate while sex was controlled for in both models, and subject ID was included as the random effect term. These were employed as following:

(2) $$\mathrm{BAG}\;AdipositymeasurexTP+Sex+Age\left(1\right|\mathrm{SubjectID})$$

(3) $$\mathrm{BAG}\;AdipositymeasurexAge+Sex+TP\left(1\right|\mathrm{SubjectID})$$

In order to prevent false positives and to regularise the estimated associations, we defined a standard prior around zero with a standard deviation of 1 for all coefficients. All variables bar sex were scaled prior to running the analyses. For each coefficient of interest, we report the mean estimated value and its uncertainty measured by the 95% credible interval (the interval in which a parameter has a given probability, considered as the equivalent to the frequentist confidence interval) of the posterior distribution, and calculated Bayes factors (BF) – provided as evidence ratios in the presented figures – using the Savage-Dickey method (Wagenmakers et al., 2010). Briefly, BF can be interpreted as a measure of the strength of evidence in favour of the null or alternative hypothesis. In the current study, values of 1 can be interpreted as no evidence in either direction, with the following values indicating weight of evidence towards the alternative hypothesis: 0.3–1 (anecdotal), 0.1–0.3 (moderate), 0.03–0.1 (strong), 0.01–0.03 (very strong), <0.01 (extreme). Contrarily, the following values indicate weight of evidence towards the null hypothesis: 1–3 (anecdotal), 3–10 (moderate), 10–30 (strong), 30–100 (very strong), >100 (extreme). For a pragmatic guide on BF interpretation, see SI Table 3.

## 3 Results

### 3.1 Brain age prediction

Age prediction accuracy in the training and test sets are summarised in SI Table 4. Briefly, the models revealed high accuracy, as previously reported (Beck et al., 2021a), with R^2^ = 0.72 and 0.73 for the T1 and DTI models, respectively. Fig. 4 shows predicted age for each model plotted as a function of age for both timepoints.

### 3.2 Adiposity measures and brain age gap

#### 3.2.1 Descriptive statistics

**Fig. 3:**
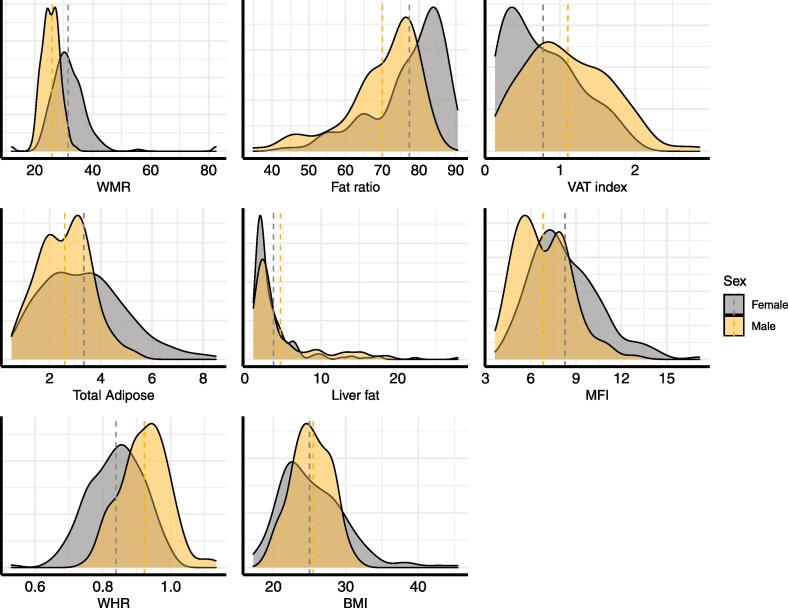
Distribution of the adiposity measures. Density plots for each variable, split by sex (male = yellow, female = grey). Vertical lines represent mean values for each sex. (For interpretation of the references to colour in this figure legend, the reader is referred to the web version of this article.)

Descriptive statistics are shown in Table 1, while Fig. 3 shows the distributions within females and males for each adiposity measure, and Fig. 5 shows the correlations between adiposity measures.

**Fig. 4:**
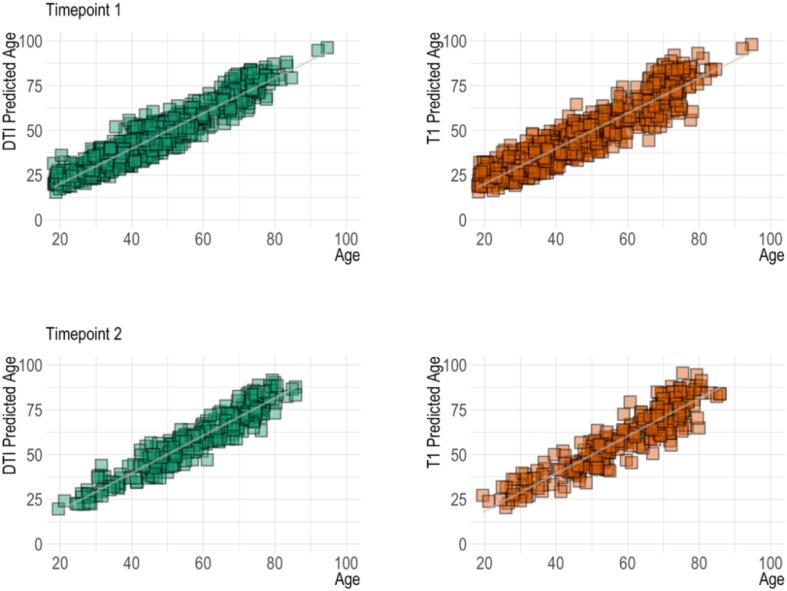
Predicted age as a function of age. For each modality, the figure shows baseline age and predicted age in the top row and follow up age and predicted age in the bottom row.

**Fig. 5:**
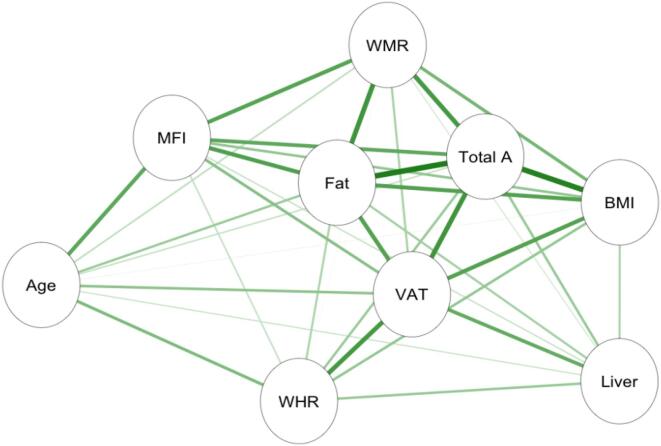
Associations between adiposity measures. Network correlation graph showing correlations between adiposity measures and age, where the green lines indicate positive associations, and orange lines (none present) indicate negative associations. Strength of association is marked by thickness of each line, with the thickest line shown equating to r = 0.86. Abbreviations: MFI – muscle fat infiltration; Fat – fat ratio; WHR – waist-to-hip ratio; VAT – visceral abdominal tissue index; WMR – weight-to-muscle ratio; Total A – total adipose; BMI – body-mass index; Liver – liver fat. (For interpretation of the references to colour in this figure legend, the reader is referred to the web version of this article.)

### 3.3 Bayesian multilevel models

#### 3.3.1 Associations between BAG and adiposity measure

**Fig. 6:**
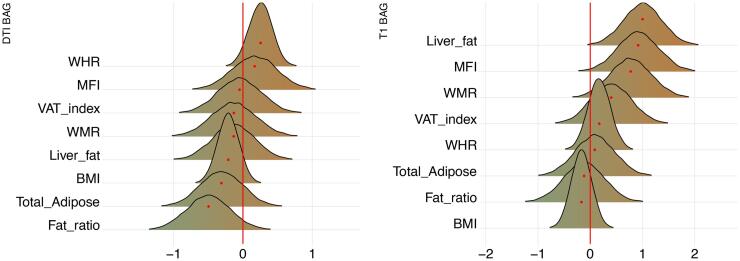
Associations between adiposity and BAG. The figure shows posterior distributions of the estimates of the standardised coefficient. Estimates for each variable on DTI BAG on the left and T1 BAG on the right. Colour scale follows direction evidence, with positive values indicating evidence in favour of an association and negative values evidence in favour of the null hypothesis. Width of distribution represents the uncertainty of the parameter estimates.

**Fig. 7:**
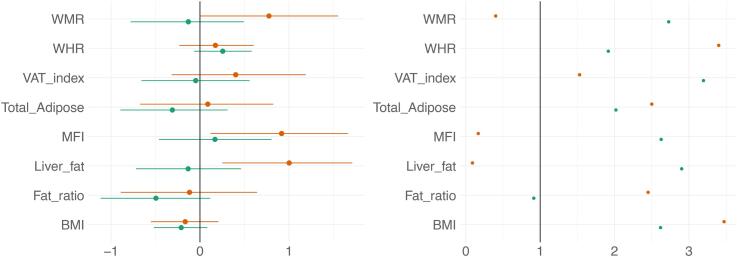
BAG and adiposity: Estimate credible intervals and evidence ratios. Left-side plot shows estimates with 95% credible intervals while the right-side figure shows likelihood of null where values above one indicate evidence in favour of the effect being null, and values below one indicate evidence in favour of an effect. T1 BAG associations are represented by orange points, and DTI BAG by green points. (For interpretation of the references to colour in this figure legend, the reader is referred to the web version of this article.)

Posterior distributions of the estimates of the coefficients reflecting the associations between each adiposity measure and BAGs are shown in Fig. 6, where parameterisation of the model can be interpreted as values increasing from 0 to 1 indicating evidence in favour of an association and values decreasing 0 to −1 indicating evidence in favour of the null hypothesis. Fig. 7 shows credible intervals and evidence ratios. SI Fig. 7 shows network correlation graph of correlations between adiposity measures and each BAG, and SI Table 5 provides summary statistics.

The tests revealed anecdotal evidence in favour of an association with DTI BAG for fat ratio (BF = 0.91, *β = -*0.50), while moderate evidence in favour of no association was revealed between DTI BAG and VAT index (BF = 3.12, *β =* -0.05), liver fat (BF = 2.90, *β =* -0.13), and anecdotal evidence for WMR (BF = 2.73, *β = -*0.13), total adipose (BF = 2.02, *β =* -0.31), MFI (BF = 2.63, *β =* 0.17), WHR (BF = 1.92, *β =* 0.26), and BMI (BF = 2.62, *β = -*0.21).

Strong evidence in favour of an association with T1 BAG was provided for liver fat (BF = 0.09, *β =* 1.0), with moderate evidence for MFI (BF = 0.17, *β =* 0.92), and anecdotal evidence for WMR (BF = 0.40, *β =* 0.77). The tests revealed moderate evidence in favour of no association between T1 BAG and WHR (BF = 3.40, *β =* 1.73), and BMI (BF = 3.47, *β =* -0.17), and anecdotal evidence for fat ratio (BF = 2.5, *β =* -0.12), total adipose (BF = 2.50, *β =* 0.09), VAT index (BF = 1.53, *β =* 0.40).

#### 3.3.2 Interaction effects of time and adiposity measure on brain age gap

**Fig. 8:**
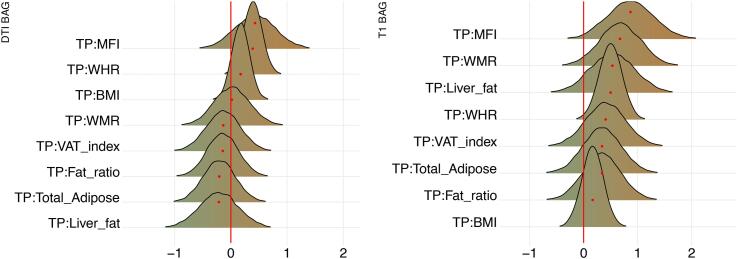
Interaction effects between adiposity and time on BAG. The figure shows posterior distributions of the estimates of the standardised coefficient. Estimates for the interaction effect of time and each adiposity measure on DTI BAG on the left and T1 BAG on the right.

**Fig. 9:**
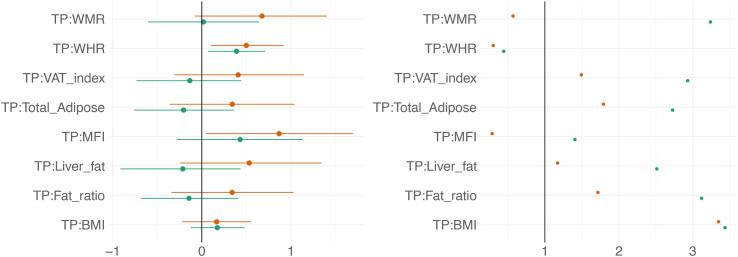
Interaction effects between adiposity and time on BAG: Estimate credible intervals and evidence ratios. Left-side plot shows estimates with 95% credible intervals while the right-side figure shows likelihood ratios. T1 BAG effects are represented by orange points, and DTI BAG by green points. (For interpretation of the references to colour in this figure legend, the reader is referred to the web version of this article.)

Posterior distributions of the estimates of the coefficient for the interaction between time and each adiposity measure and DTI and T1 BAGs are shown in Fig. 8, and Fig. 9 shows credible intervals and evidence ratios. For full table of results see SI Table 5. For supplementary visualisation of longitudinal results, see SI Figs. 8 and 9.

For DTI BAG, the evidence supporting an interaction with time was anecdotal for WHR (BF = 0.44, *β =* 0.39). The models revealed moderate evidence in favour of no interaction with time for WMR (BF = 3.24, *β =* 0.02), fat ratio (BF = 3.12, *β =* -0.14), and BMI (BF = 3.43, *β =* 0.17), with anecdotal evidence for VAT index (BF = 2.93, *β =* -0.13), total adipose (BF = 2.73, *β =* -0.21), liver fat (BF = 2.51, *β =* -0.21), and MFI (BF = 1.41, *β =* 0.43).

For T1 BAG, the evidence supporting an interaction with time was moderate for WHR (BF = 0.30, *β =* 0.50) and MFI (BF = 0.29, *β =* 0.87), indicating faster pace of brain ageing among people with higher WHR and MFI. The models also revealed anecdotal evidence for WMR (BF = 0.57, *β =* 0.66). The evidence of no associations was anecdotal for fat ratio (BF = 1.72, *β =* 0.34), VAT index (BF = 1.49, *β =* 0.41), total adipose (BF = 1.79, *β =* 0.34), and liver fat (BF = 1.17, *β =* 0.53), and moderate for BMI (BF = 3.35, *β =* 0.17).

#### 3.3.3 Interaction effects of age and adiposity measure on brain age gap

**Fig. 10:**
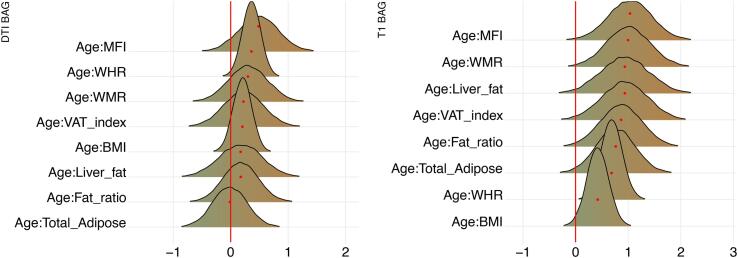
Interaction effects between adiposity and age on BAG. The figure shows posterior distributions of the estimates for the interaction effect between age and each variable on DTI BAG on the left and T1 BAG on the right.

**Fig. 11:**
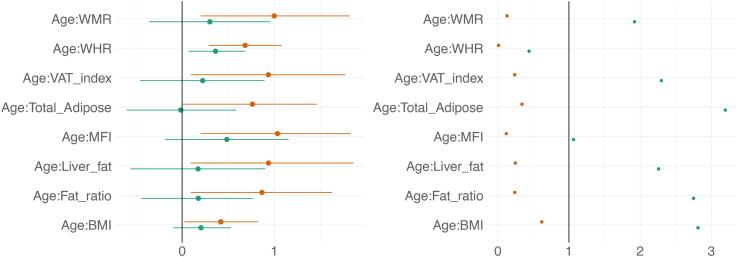
Interaction effects between adiposity and age on BAG: Estimate credible intervals and evidence ratios. Left-side plot shows estimates with 95% credible intervals while the right-side figure shows likelihood ratios. T1 BAG effects are represented by orange points, and DTI BAG by green points. (For interpretation of the references to colour in this figure legend, the reader is referred to the web version of this article.)

Posterior distributions of the estimates of the coefficient for the interaction between age and each adiposity measure and DTI and T1 BAGs are shown in Fig. 10, and Fig. 11 shows credible intervals and evidence ratios. For full table of reported results see SI Table 5.

The analysis provided anecdotal evidence in support of an interaction effect with age on DTI BAG for WHR (BF = 0.44, *β =* 0.36). Anecdotal evidence was also provided in support of no interaction effect for WMR (BF = 1.92, *β =* 0.30), liver fat (BF = 2.26, *β =* 0.17), MFI (BF = 1.06, *β =* 0.49), fat ratio (BF = 2.75, *β =* 0.18), VAT index (BF = 2.29, *β =* 0.22), and BMI (BF = 2.81, *β =* 0.21), with moderate evidence for total adipose (BF = 3.20, *β =* -0.01).

The support of an interaction effect with age on T1 BAG was very strong for WHR (BF = 0.01, *β =* 0.68), and moderate for WMR (BF = 0.13, *β =* 1.00), fat ratio (BF = 0.24, *β =* 0.87), VAT index (BF = 0.24, *β =* 0.94), liver fat (BF = 0.25, *β =* 0.94), and MFI (BF = 0.12, *β =* 1.03), indicating these adiposity measures may be increasingly important predictors of BAG with increasing age. The models further indicated anecdotal evidence for total adipose (BF = 0.34, *β =* 0.76) and BMI (BF = 0.62, *β =* 0.42).

## 4 Discussion

Despite increasing evidence of shared mechanisms across several metabolic conditions and cardiovascular and neurodegenerative diseases, we are yet to fully understand the complex associations of adipose tissue and brain age. The current cross-sectional and longitudinal findings support that higher measures of adipose tissue – particularly higher liver fat and MFI – are associated with an older-appearing brain and faster brain ageing. Both overall brain age gap and the rates of change in brain age over time were associated with specific adipose tissue measures at follow-up, including thigh muscle fat infiltration, weight-to-muscle ratio, and liver fat.

### 4.1 Adiposity and brain age: cross-sectional analysis

Our findings demonstrated associations between liver fat, MFI, WMR and T1 BAG, indicating older-appearing brains in individuals with higher adipose tissue measures and supporting our hypotheses for these specific measurements. Conversely, our hypotheses for fat ratio, total adipose, and VAT index were not supported. DTI BAG associations were less prevalent, with anecdotal evidence in favour of an association for fat ratio, while moderate evidence in favour of no association was revealed for VAT index and liver fat, meaning only our hypothesis for fat ratio was supported by the results. Our hypothesis that stronger associations would be found for body MRI measures than for traditional anthropometric features was supported by results pertaining to T1 BAG, however, for DTI BAG, anthropometric features performed comparably well to body MRI measures.

The findings are in line with the hypothesis that body MRI adipose tissue measures are associated with ageing of the brain as indicated by brain MRI morphology measures. Moreover, the findings extend previous work linking adiposity measures, including links to grey matter volume (Debette and Markus, 2010, Gunstad et al., 2005, Jørgensen et al., 2017, Ward et al., 2005) and white matter microstructural properties based on diffusion MRI (Marks et al., 2011, Mueller et al., 2011, Stanek et al., 2011, Xu et al., 2013), with conflicting evidence for white matter volume (Friedman et al., 2014, Willette and Kapogiannis, 2015). The discrepancy may be due to methodological differences, e.g., between previous regional associations and our global brain age approach. Future research estimating regional brain age models trained with more advanced diffusion MRI features may offer improved sensitivity and specificity (Beck et al., 2021b).

MFI has previously been linked to metabolic risk factors (Therkelsen et al., 2013) and insulin resistance in obesity (Goodpaster et al., 2000). Higher liver fat has been found among diabetics (Bamberg et al., 2017, Linge et al., 2018) and prediabetics without previous cardiovascular conditions (Bamberg et al., 2017). Studies have also reported no significant association between CHD or cardiovascular events and elevated liver fat (Neeland et al., 2015). Moreover, while comparing patients with non-alcoholic fatty liver disease (NAFLD) to controls, Hagström et al., (2017) reported no significant difference in cardiovascular related death. Conflicting results however, have linked NAFLD with cardiovascular disease (Brea et al., 2017), suggesting a complex interplay between regional adipose tissue and metabolic health.

This complexity has recently been corroborated by findings of 62 genetic loci associated with both higher adiposity and lower cardiometabolic risk (Huang et al., 2021), possibly reflecting protective mechanisms. Although speculative, this heterogeneity is likely also reflected in connection to brain health, which for some individual genetic architectures may offer protection against brain pathology while simultaneously increasing the risk for obesity. Further studies exploiting larger sampler are needed to pursue this hypothesis and attempt to dissect the heterogeneity using genetic data (Huang et al., 2021) in combination with brain imaging.

### 4.2 Adiposity and brain ageing: Longitudinal evidence

Our longitudinal analyses revealed that the rate of brain ageing across the study period was associated with adiposity measured at follow-up, with evidence for WHR from our anthropometric measures, and MFI and WMR from adipose tissue distribution measures. While evidence for WHR was present for both T1-weighted and DTI BAGs – which largely replicates a recent report from the same cohort (Beck et al., 2021a) – evidence for MFI and WMR were only observed for T1-weighted BAG, where greater adipose tissue was associated with increased rate of brain ageing. Conversely, evidence in favour of no interaction effect with time was found for BMI. This supports that BMI alone may not represent a clinically relevant proxy, as it fails to distinguish from lean mass while ignoring regional fat distribution, in particular the visceral components (Evans et al., 2012, Roberson et al., 2014).

While experimental evidence is needed to establish causality, our findings suggest that adipose tissue distribution affects brain ageing and may lead to older appearing brains in generally healthy individuals. These observations are in line with previous studies, both from clinical groups and population-based cohorts. NADLD, for example, has been previously linked to smaller total brain volume (Weinstein et al., 2018), and liver fat has been linked to smaller cortical and cerebellar structures (Gurholt et al., 2020). Moreover, negative associations have been reported between MFI and cortical structures (Gurholt et al., 2020). While more research into adipose tissue distribution is warranted, the current results suggest that increased liver fat, muscle fat infiltration, and weight-to-muscle ratio may contribute to accelerate brain ageing.

### 4.3 Brain age and adiposity: interactions with age

For DTI BAG, our findings demonstrated an interaction effect with age and WHR, while T1 BAG interaction effects were present for age and WMR, fat ratio, total adipose, VAT index, liver fat, MFI, BMI, and WHR. The findings indicate that adiposity measures may be increasingly important predictors of BAG with increasing age. In contrast, we observed no evidence of interaction effects between age and BMI on DTI or T1 BAGs. Previous research has produced mixed results, with studies reporting an interaction of BMI and age on white matter volume but no interaction on cortical surface area or thickness (Ronan et al., 2016). Further research is warranted to elucidate the degree to which associations between adiposity and brain structure change over the course of the lifespan.

### 4.4 Strengths and limitations

The current study benefitted from a mixed cross-sectional and longitudinal design enabling brain changes to be tracked across time. The prediction models for brain age had high accuracy, and separate T1-weighted and DTI brain age gaps provided tissue-specific measures of brain age with potential to reveal specific associations with the included adiposity measures.

Several limitations should be considered when evaluating the findings. First, while the longitudinal brain MRI data represent a major strength, the follow-up sample size is relatively small, and the body MRI data was collected at the follow-up examination only. The subsequent loss of power is reflected in the width of body MRI posterior distributions, indicating a higher level of uncertainty compared to BMI and WHR, which had available longitudinal measures and a larger sample size. Next, although body composition measures based on MRI offer high accuracy in terms of fat and muscle distributions and are therefore a potentially valuable supplement to conventional anthropometric features, future studies including biological markers such as immune and inflammation assays and lipid measurements might provide even higher specificity and opportunities for further subtyping. Indeed, inflammation has demonstrated effects on brain function and structure (Rosano et al., 2011) and has been dubbed to have a central role in the obesity-brain connection (O’Brien et al., 2017).

Further, there is the possibility of transfer learning happening and scanner effects inducing an offset shift in the estimates, however, the current study utilises the relative estimates within the test set rather than the absolute age estimates and thus this should not introduce a problem for the conclusions drawn. Moreover, including detailed assessments of dietary routines, alcohol intake, and physical exercise is vital in order to better understand the complex processes at play. For example, physical activity has been associated with lower brain age (Dunås et al., 2021, Sanders et al., 2021) and higher grey and white matter measures (Sexton et al., 2016), while excess alcohol intake is well documented in influencing liver and brain health (Agartz et al., 1999, Rehm et al., 2010). Lastly, the current sample is predominantly ethnic Scandinavian and Northern European, restricting our ability to generalise to wider populations, and future studies should aim to increase the diversity in the study population.

### 4.5 Conclusion

Combining body MRI and brain age prediction based on brain MRI allows for probing individual body composition profiles and brain patterns and trajectories which may confer risk for cardiometabolic disease and neurodegenerative disorders. More knowledge and further development of automated tools for individual phenotyping in this domain may inform public health priorities and interventions. With evidence of different adiposity subtypes being differentially linked with different brain phenotypes and cardiometabolic diseases, precision methods that look at fat distribution can potentially be more informative than conventional anthropometric measures. This in turn will provide a more effective tool for development of treatment strategies that focus on individual risk of metabolic disease, as well as disentangling the associations between body and brain health.

#### CRediT authorship contribution statement

**Dani Beck:** Conceptualization, Writing – original draft, Writing – review & editing, Investigation, Software, Visualization, Formal analysis, Project administration. **Ann-Marie G. de Lange:** Supervision, Writing – review & editing, Formal analysis, Funding acquisition. **Dag Alnæs:** Methodology, Data curation, Writing – review & editing. **Ivan I. Maximov:** Data curation, Writing – review & editing. **Mads L. Pedersen:** Formal analysis, Visualization, Writing – review & editing. **Olof Dahlqvist Leinhard:** Writing – review & editing. **Jennifer Linge:** Writing – review & editing. **Rozalyn Simon:** Writing – review & editing. **Geneviève Richard:** Project administration, Investigation, Writing – review & editing. **Kristine M. Ulrichsen:** Project administration, Investigation, Writing – review & editing. **Erlend S. Dørum:** Project administration, Investigation, Writing – review & editing. **Knut K. Kolskår:** Project administration, Investigation, Writing – review & editing. **Anne-Marthe Sanders:** Project administration, Investigation, Writing – review & editing. **Adriano Winterton:** Writing – review & editing. **Tiril P. Gurholt:** Writing – review & editing. **Tobias Kaufmann:** Funding acquisition, Writing – review & editing. **Nils Eiel Steen:** Writing – review & editing. **Jan Egil Nordvik:** Funding acquisition, Writing – review & editing. **Ole A. Andreassen:** Funding acquisition, Writing – review & editing, Investigation. **Lars T. Westlye:** Conceptualization, Writing – review & editing, Supervision, Funding acquisition.

## Declaration of Competing Interest

The authors declare that they have no known competing financial interests or personal relationships that could have appeared to influence the work reported in this paper.
