# Supplementary material for: Adipose tissue distribution from body MRI is associated with cross-sectional and longitudinal brain age in adults
Source: Neuroimage Clin. 2022 Jan 27;33:102949. doi: 10.1016/j.nicl.2022.102949 (PMC8814666; doi:10.1016/j.nicl.2022.102949)
Supplement: Supplementary Data 1 [file mmc1.docx]

**Supplementary Material**

| **SI Table 1.** Overview of DTI (276) and T1-weighted (269) features used in the prediction model. For DTI, FA = fractional anisotropy, MD = mean diffusivity, L1 = Axial diffusivity, and RD = radial diffusivity. | |
| --- | --- |
| **DTI** | FA_ATRL FA_ATRR FA_CSTL FA_CSTR FA_CGL FA_CGR FA_CINGHL FA_CINGHR FA_FMAJ FA_FMIN FA_IFOFL FA_IFOFR FA_ILFL FA_ILFR FA_SLFL FA_SLFR FA_UFL FA_UFR FA_SLFTL FA_SLTFR FA_Middlecerebellarpeduncle FA_Pontine FA_GenuCC FA_BodyCC FA_SpleniumCC FA_Fornix FA_CorticospinaltractR FA_CorticospinaltractL FA_MediallemniscusR FA_MediallemniscusL FA_InferiorcerebellarpeduncleR FA_InferiorcerebellarpeduncleL FA_SuperiorcerebellarpeduncleR FA_SuperiorcerebellarpeduncleL FA_CerebralpeduncleR FA_CerebralpeduncleL FA_AnteriorlimbofinternalcapsuleR FA_AnteriorlimbofinternalcapsuleL FA_PosteriorlimbofinternalcapsuleR FA_PosteriorlimbofinternalcapsuleL FA_RetrolenticularpartofinternalcapsuleR FA_RetrolenticularpartofinternalcapsuleL FA_AnteriorcoronaradiataR FA_AnteriorcoronaradiataL FA_SuperiorcoronaradiataR FA_SuperiorcoronaradiataL FA_PosteriorcoronaradiataR FA_PosteriorcoronaradiataL FA_PosteriorthalamicradiationR FA_PosteriorthalamicradiationL FA_SagittalstratumR FA_SagittalstratumL FA_ExternalcapsuleR FA_ExternalcapsuleL FA_CingulumcingulategyrusR FA_CingulumcingulategyrusL FA_CingulumhippocampusR FA_CingulumhippocampusL FA_FornixStriaterminalisR FA_FornixStriaterminalisL FA_SuperiorlongitudinalfasciculusR FA_SuperiorlongitudinalfasciculusL FA_SuperiorfrontooccipitalfasciculusR FA_SuperiorfrontooccipitalfasciculusL FA_UncinatefasciculusR FA_UncinatefasciculusL FA_TapetumR FA_TapetumL FA_meanskel MD_ATRL MD_ATRR MD_CSTL MD_CSTR MD_CGL MD_CGR MD_CINGHL MD_CINGHR MD_FMAJ MD_FMIN MD_IFOFL MD_IFOFR MD_ILFL MD_ILFR MD_SLFL MD_SLFR MD_UFL MD_UFR MD_SLFTL MD_SLTFR MD_Middlecerebellarpeduncle MD_Pontine MD_GenuCC MD_BodyCC MD_SpleniumCC MD_Fornix MD_CorticospinaltractR MD_CorticospinaltractL MD_MediallemniscusR MD_MediallemniscusL MD_InferiorcerebellarpeduncleR MD_InferiorcerebellarpeduncleL MD_SuperiorcerebellarpeduncleR MD_SuperiorcerebellarpeduncleL MD_CerebralpeduncleR MD_CerebralpeduncleL MD_AnteriorlimbofinternalcapsuleR MD_AnteriorlimbofinternalcapsuleL MD_PosteriorlimbofinternalcapsuleR MD_PosteriorlimbofinternalcapsuleL MD_RetrolenticularpartofinternalcapsuleR MD_RetrolenticularpartofinternalcapsuleL MD_AnteriorcoronaradiataR MD_AnteriorcoronaradiataL MD_SuperiorcoronaradiataR MD_SuperiorcoronaradiataL MD_PosteriorcoronaradiataR MD_PosteriorcoronaradiataL MD_PosteriorthalamicradiationR MD_PosteriorthalamicradiationL MD_SagittalstratumR MD_SagittalstratumL MD_ExternalcapsuleR MD_ExternalcapsuleL MD_CingulumcingulategyrusR MD_CingulumcingulategyrusL MD_CingulumhippocampusR MD_CingulumhippocampusL MD_FornixStriaterminalisR MD_FornixStriaterminalisL MD_SuperiorlongitudinalfasciculusR MD_SuperiorlongitudinalfasciculusL MD_SuperiorfrontooccipitalfasciculusR MD_SuperiorfrontooccipitalfasciculusL MD_UncinatefasciculusR MD_UncinatefasciculusL MD_TapetumR MD_TapetumL MD_meanskel L1_ATRL L1_ATRR L1_CSTL L1_CSTR L1_CGL L1_CGR L1_CINGHL L1_CINGHR L1_FMAJ L1_FMIN L1_IFOFL L1_IFOFR L1_ILFL L1_ILFR L1_SLFL L1_SLFR L1_UFL L1_UFR L1_SLFTL L1_SLTFR L1_Middlecerebellarpeduncle L1_Pontine L1_GenuCC L1_BodyCC L1_SpleniumCC L1_Fornix L1_CorticospinaltractR L1_CorticospinaltractL L1_MediallemniscusR L1_MediallemniscusL L1_InferiorcerebellarpeduncleR L1_InferiorcerebellarpeduncleL L1_SuperiorcerebellarpeduncleR L1_SuperiorcerebellarpeduncleL L1_CerebralpeduncleR L1_CerebralpeduncleL L1_AnteriorlimbofinternalcapsuleR L1_AnteriorlimbofinternalcapsuleL L1_PosteriorlimbofinternalcapsuleR L1_PosteriorlimbofinternalcapsuleL L1_RetrolenticularpartofinternalcapsuleR L1_RetrolenticularpartofinternalcapsuleL L1_AnteriorcoronaradiataR L1_AnteriorcoronaradiataL L1_SuperiorcoronaradiataR L1_SuperiorcoronaradiataL L1_PosteriorcoronaradiataR L1_PosteriorcoronaradiataL L1_PosteriorthalamicradiationR L1_PosteriorthalamicradiationL L1_SagittalstratumR L1_SagittalstratumL L1_ExternalcapsuleR L1_ExternalcapsuleL L1_CingulumcingulategyrusR L1_CingulumcingulategyrusL L1_CingulumhippocampusR L1_CingulumhippocampusL L1_FornixStriaterminalisR L1_FornixStriaterminalisL L1_SuperiorlongitudinalfasciculusR L1_SuperiorlongitudinalfasciculusL L1_SuperiorfrontooccipitalfasciculusR L1_SuperiorfrontooccipitalfasciculusL L1_UncinatefasciculusR L1_UncinatefasciculusL L1_TapetumR L1_TapetumL L1_meanskel RD_ATRL RD_ATRR RD_CSTL RD_CSTR RD_CGL RD_CGR RD_CINGHL RD_CINGHR RD_FMAJ RD_FMIN RD_IFOFL RD_IFOFR RD_ILFL RD_ILFR RD_SLFL RD_SLFR RD_UFL RD_UFR RD_SLFTL RD_SLTFR RD_Middlecerebellarpeduncle RD_Pontine RD_GenuCC RD_BodyCC RD_SpleniumCC RD_Fornix RD_CorticospinaltractR RD_CorticospinaltractL RD_MediallemniscusR RD_MediallemniscusL RD_InferiorcerebellarpeduncleR RD_InferiorcerebellarpeduncleL RD_SuperiorcerebellarpeduncleR RD_SuperiorcerebellarpeduncleL RD_CerebralpeduncleR RD_CerebralpeduncleL RD_AnteriorlimbofinternalcapsuleR RD_AnteriorlimbofinternalcapsuleL RD_PosteriorlimbofinternalcapsuleR RD_PosteriorlimbofinternalcapsuleL RD_RetrolenticularpartofinternalcapsuleR RD_RetrolenticularpartofinternalcapsuleL RD_AnteriorcoronaradiataR RD_AnteriorcoronaradiataL RD_SuperiorcoronaradiataR RD_SuperiorcoronaradiataL RD_PosteriorcoronaradiataR RD_PosteriorcoronaradiataL RD_PosteriorthalamicradiationR RD_PosteriorthalamicradiationL RD_SagittalstratumR RD_SagittalstratumL RD_ExternalcapsuleR RD_ExternalcapsuleL RD_CingulumcingulategyrusR RD_CingulumcingulategyrusL RD_CingulumhippocampusR RD_CingulumhippocampusL RD_FornixStriaterminalisR RD_FornixStriaterminalisL RD_SuperiorlongitudinalfasciculusR RD_SuperiorlongitudinalfasciculusL RD_SuperiorfrontooccipitalfasciculusR RD_SuperiorfrontooccipitalfasciculusL RD_UncinatefasciculusR RD_UncinatefasciculusL RD_TapetumR RD_TapetumL RD_meanskel |
| **T1** | lh_bankssts_thickness lh_caudalanteriorcingulate_thickness lh_caudalmiddlefrontal_thickness lh_cuneus_thickness lh_entorhinal_thickness lh_fusiform_thickness lh_inferiorparietal_thickness lh_inferiortemporal_thickness lh_isthmuscingulate_thickness lh_lateraloccipital_thickness lh_lateralorbitofrontal_thickness lh_lingual_thickness lh_medialorbitofrontal_thickness lh_middletemporal_thickness lh_parahippocampal_thickness lh_paracentral_thickness lh_parsopercularis_thickness lh_parsorbitalis_thickness lh_parstriangularis_thickness lh_pericalcarine_thickness lh_postcentral_thickness lh_posteriorcingulate_thickness lh_precentral_thickness lh_precuneus_thickness lh_rostralanteriorcingulate_thickness lh_rostralmiddlefrontal_thickness lh_superiorfrontal_thickness lh_superiorparietal_thickness lh_superiortemporal_thickness lh_supramarginal_thickness lh_frontalpole_thickness lh_temporalpole_thickness lh_transversetemporal_thickness lh_insula_thickness lh_MeanThickness_thickness rh_bankssts_thickness rh_caudalanteriorcingulate_thickness rh_caudalmiddlefrontal_thickness rh_cuneus_thickness rh_entorhinal_thickness rh_fusiform_thickness rh_inferiorparietal_thickness rh_inferiortemporal_thickness rh_isthmuscingulate_thickness rh_lateraloccipital_thickness rh_lateralorbitofrontal_thickness rh_lingual_thickness rh_medialorbitofrontal_thickness rh_middletemporal_thickness rh_parahippocampal_thickness rh_paracentral_thickness rh_parsopercularis_thickness rh_parsorbitalis_thickness rh_parstriangularis_thickness rh_pericalcarine_thickness rh_postcentral_thickness rh_posteriorcingulate_thickness rh_precentral_thickness rh_precuneus_thickness rh_rostralanteriorcingulate_thickness rh_rostralmiddlefrontal_thickness rh_superiorfrontal_thickness rh_superiorparietal_thickness rh_superiortemporal_thickness rh_supramarginal_thickness rh_frontalpole_thickness rh_temporalpole_thickness rh_transversetemporal_thickness rh_insula_thickness rh_MeanThickness_thickness lh_bankssts_volume lh_caudalanteriorcingulate_volume lh_caudalmiddlefrontal_volume lh_cuneus_volume lh_entorhinal_volume lh_fusiform_volume lh_inferiorparietal_volume lh_inferiortemporal_volume lh_isthmuscingulate_volume lh_lateraloccipital_volume lh_lateralorbitofrontal_volume lh_lingual_volume lh_medialorbitofrontal_volume lh_middletemporal_volume lh_parahippocampal_volume lh_paracentral_volume lh_parsopercularis_volume lh_parsorbitalis_volume lh_parstriangularis_volume lh_pericalcarine_volume lh_postcentral_volume lh_posteriorcingulate_volume lh_precentral_volume lh_precuneus_volume lh_rostralanteriorcingulate_volume lh_rostralmiddlefrontal_volume lh_superiorfrontal_volume lh_superiorparietal_volume lh_superiortemporal_volume lh_supramarginal_volume lh_frontalpole_volume lh_temporalpole_volume lh_transversetemporal_volume lh_insula_volume rh_bankssts_volume rh_caudalanteriorcingulate_volume rh_caudalmiddlefrontal_volume rh_cuneus_volume rh_entorhinal_volume rh_fusiform_volume rh_inferiorparietal_volume rh_inferiortemporal_volume rh_isthmuscingulate_volume rh_lateraloccipital_volume rh_lateralorbitofrontal_volume rh_lingual_volume rh_medialorbitofrontal_volume rh_middletemporal_volume rh_parahippocampal_volume rh_paracentral_volume rh_parsopercularis_volume rh_parsorbitalis_volume rh_parstriangularis_volume rh_pericalcarine_volume rh_postcentral_volume rh_posteriorcingulate_volume rh_precentral_volume rh_precuneus_volume rh_rostralanteriorcingulate_volume rh_rostralmiddlefrontal_volume rh_superiorfrontal_volume rh_superiorparietal_volume rh_superiortemporal_volume rh_supramarginal_volume rh_frontalpole_volume rh_temporalpole_volume rh_transversetemporal_volume rh_insula_volume lh_bankssts_area lh_caudalanteriorcingulate_area lh_caudalmiddlefrontal_area lh_cuneus_area lh_entorhinal_area lh_fusiform_area lh_inferiorparietal_area lh_inferiortemporal_area lh_isthmuscingulate_area lh_lateraloccipital_area lh_lateralorbitofrontal_area lh_lingual_area lh_medialorbitofrontal_area lh_middletemporal_area lh_parahippocampal_area lh_paracentral_area lh_parsopercularis_area lh_parsorbitalis_area lh_parstriangularis_area lh_pericalcarine_area lh_postcentral_area lh_posteriorcingulate_area lh_precentral_area lh_precuneus_area lh_rostralanteriorcingulate_area lh_rostralmiddlefrontal_area lh_superiorfrontal_area lh_superiorparietal_area lh_superiortemporal_area lh_supramarginal_area lh_frontalpole_area lh_temporalpole_area lh_transversetemporal_area lh_insula_area lh_WhiteSurfArea_area rh_bankssts_area rh_caudalanteriorcingulate_area rh_caudalmiddlefrontal_area rh_cuneus_area rh_entorhinal_area rh_fusiform_area rh_inferiorparietal_area rh_inferiortemporal_area rh_isthmuscingulate_area rh_lateraloccipital_area rh_lateralorbitofrontal_area rh_lingual_area rh_medialorbitofrontal_area rh_middletemporal_area rh_parahippocampal_area rh_paracentral_area rh_parsopercularis_area rh_parsorbitalis_area rh_parstriangularis_area rh_pericalcarine_area rh_postcentral_area rh_posteriorcingulate_area rh_precentral_area rh_precuneus_area rh_rostralanteriorcingulate_area rh_rostralmiddlefrontal_area rh_superiorfrontal_area rh_superiorparietal_area rh_superiortemporal_area rh_supramarginal_area rh_frontalpole_area rh_temporalpole_area rh_transversetemporal_area rh_insula_area rh_WhiteSurfArea_area Left-Lateral-Ventricle Left-Inf-Lat-Vent Left-Cerebellum-White-Matter Left-Cerebellum-Cortex Left-Thalamus Left-Caudate Left-Putamen Left-Pallidum 3rd-Ventricle 4th-Ventricle Brain-Stem Left-Hippocampus Left-Amygdala CSF Left-Accumbens-area Left-VentralDC Left-vessel Left-choroid-plexus Right-Lateral-Ventricle Right-Inf-Lat-Vent Right-Cerebellum-White-Matter Right-Cerebellum-Cortex Right-Thalamus Right-Caudate Right-Putamen Right-Pallidum Right-Hippocampus Right-Amygdala Right-Accumbens-area Right-VentralDC Right-vessel Right-choroid-plexus 5th-Ventricle WM-hypointensities Left-WM-hypointensities Right-WM-hypointensities non-WM-hypointensities Left-non-WM-hypointensities Right-non-WM-hypointensities Optic-Chiasm CC_Posterior CC_Mid_Posterior CC_Central CC_Mid_Anterior CC_Anterior BrainSegVol BrainSegVolNotVent lhCortexVol rhCortexVol CortexVol lhCerebralWhiteMatterVol rhCerebralWhiteMatterVol CerebralWhiteMatterVol SubCortGrayVol TotalGrayVol SupraTentorialVol SupraTentorialVolNotVent MaskVol BrainSegVol-to-eTIV MaskVol-to-eTIV EstimatedTotalIntraCranialVol |
|  | |

| **SI table 2.** Overview of Quality Assurance (QA) metrics for DTI data | |
| --- | --- |
| **QA metric abbreviation** | **Measure** |
| tsnr | Temporal-signal-to-noise-ratio |
| gmean | Global mean intensity |
| drift | Linear drift of signal over time |
| outmax | Outlier measurement maximum |
| outmean | Outlier measurement average |
| meanABSrmsb | Average absolute root-mean square |
| meanRELrmsb | Average relative root-mean square |
| maxABSrmsb | Maximum absolute root-mean square |
| maxRELrmsb | Maximum relative root-mean square |
|  |  |


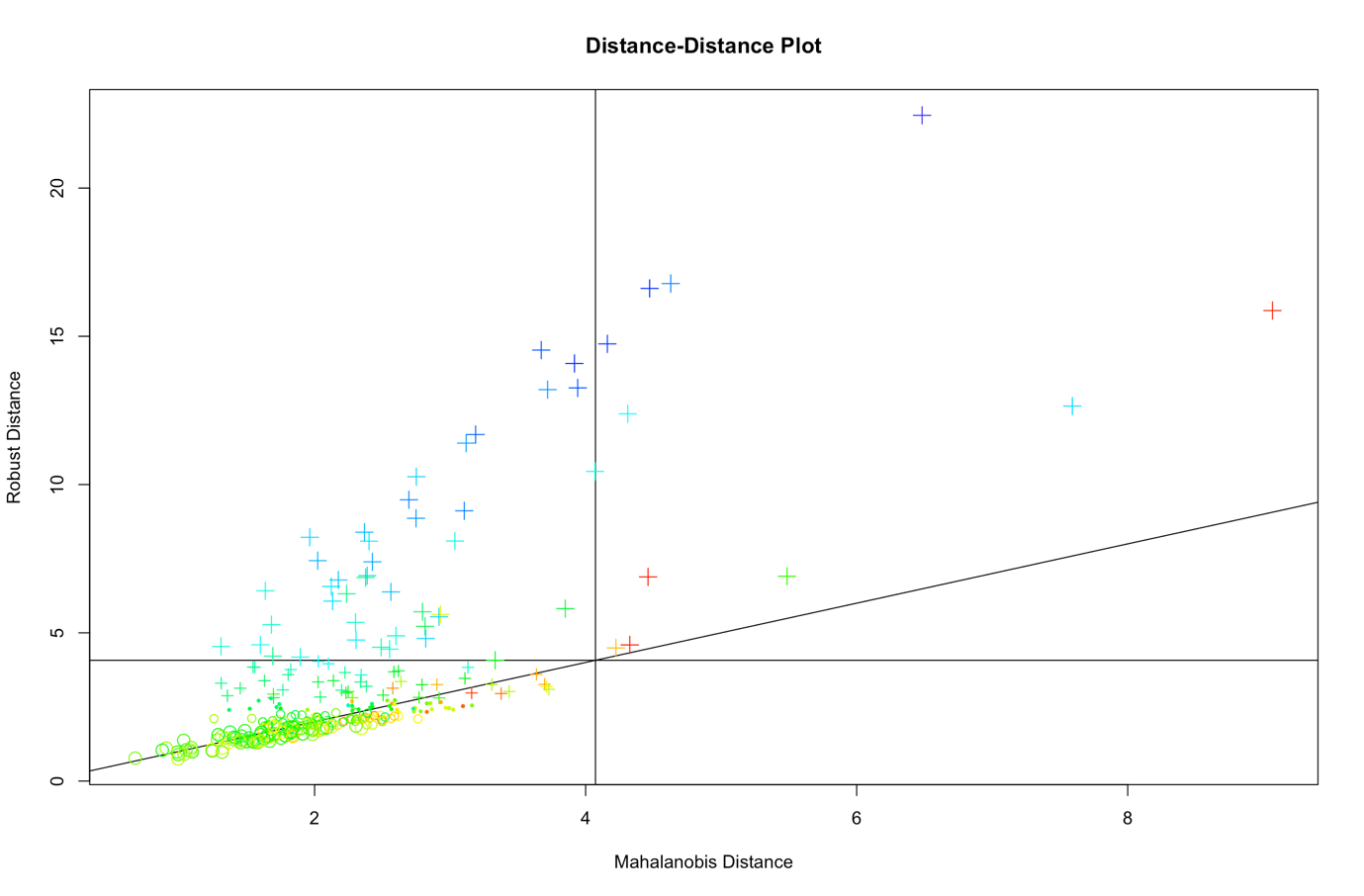


**SI Figure 1. Multivariate outlier detection algorithm.** Showing the Mahalanobis distance plot, which measures the distance between a point and a distribution to which that point belongs.


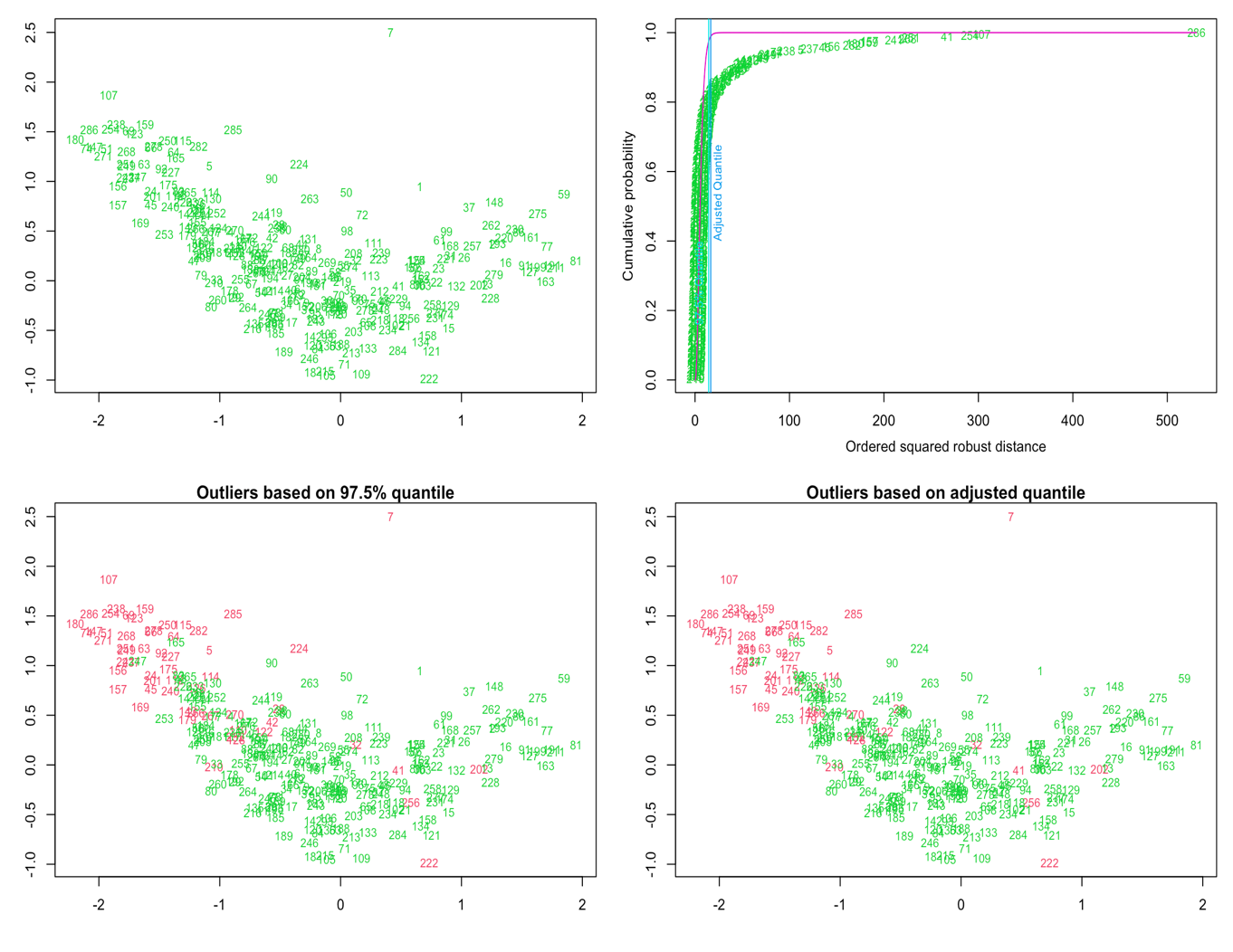


**SI Figure 2. Quantile and adjusted quantile.** Showing quantile plots which will solve for and order the squared Mahalanobis Distances for the given observations and plot them against the empirical Chi-Squared distribution function of these values. Every observation outside of the Chi-Square quantile is coloured in red.


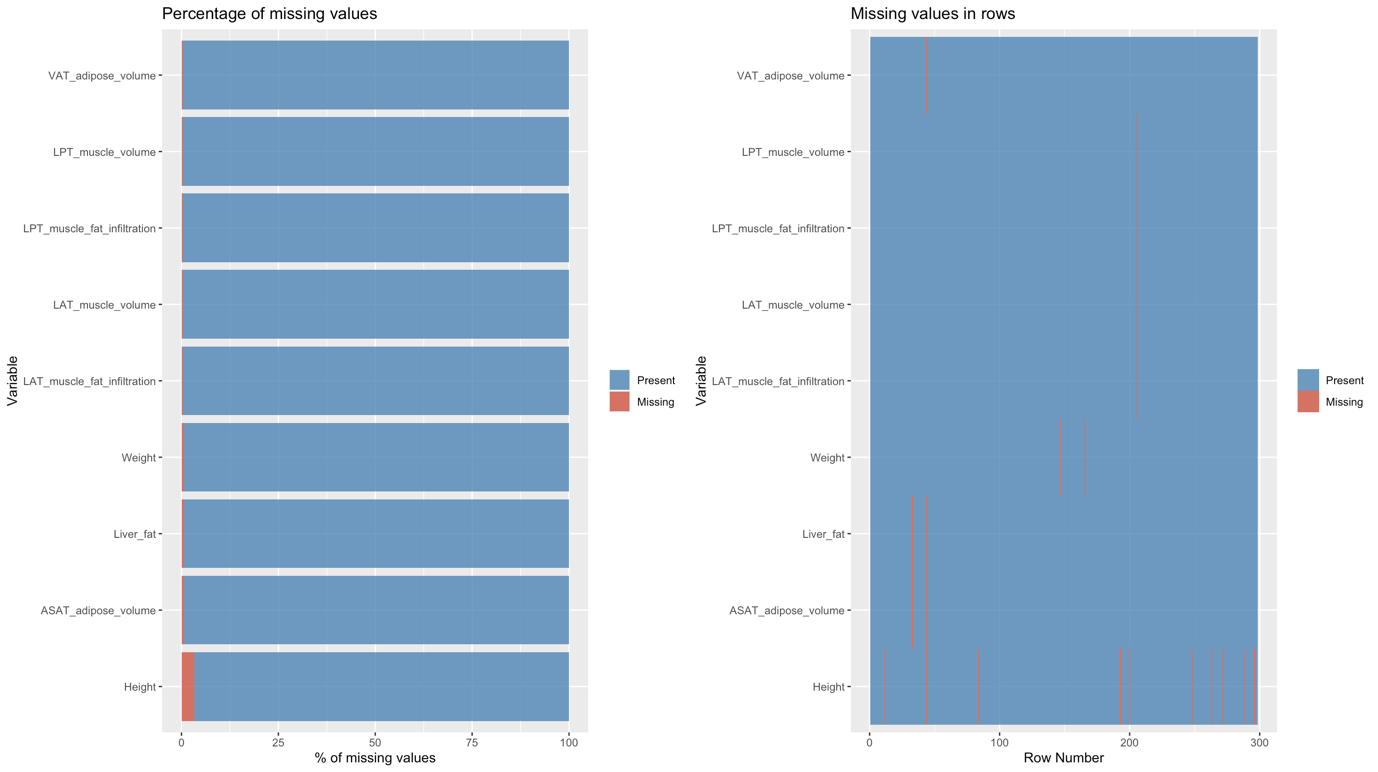


**SI Figure 3. Missing data report.** Showing percentage of missing data (left) and placement of missing data (right) for each adiposity measure prior to imputation. Of the included participants, missing entries included two ASAT, ten height, one left anterior thigh muscle fat infiltration, one left anterior thigh muscle volume, two liver fat, one left posterior thigh muscle fat infiltration, one left posterior thigh muscle volume, and two weight.


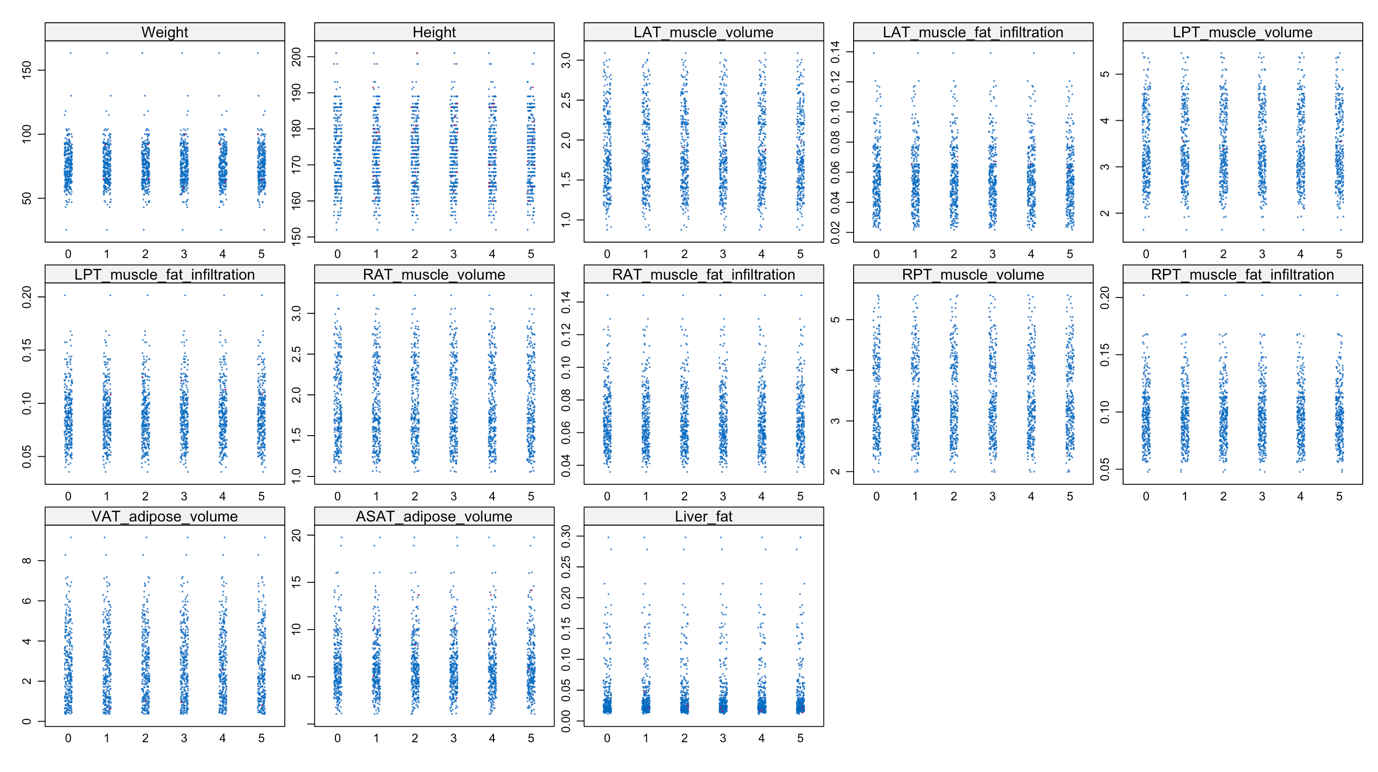


**SI Figure 4. MICE imputation.** Showing strip plot of imputed adiposity measures for five separate imputations. Blue dots represent original data, red dots represent imputed data.


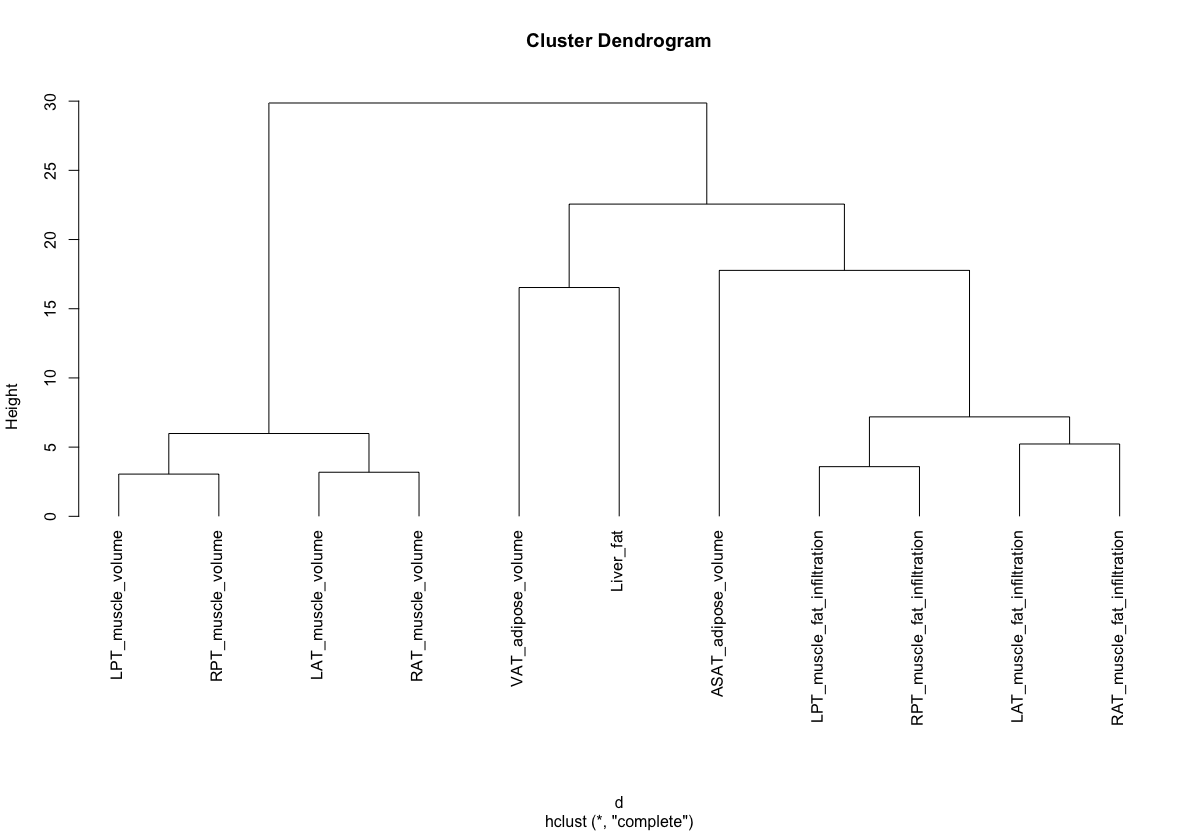
 **SI Figure 5. Hierarchical clustering.** Showing results of ‘hclust’ using the complete linkage method to form hierarchical clusters.


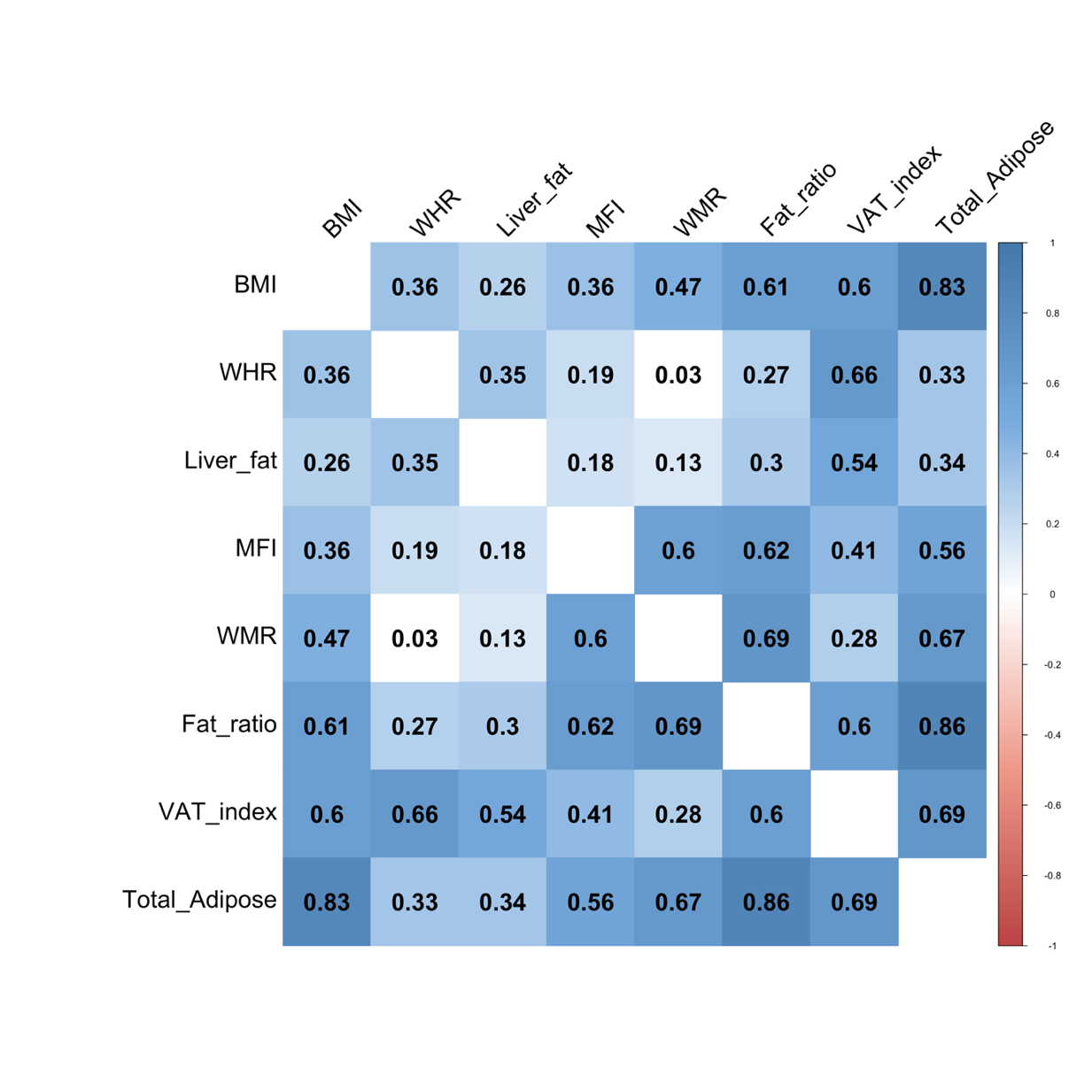


**SI Figure 6. Adiposity measure relatedness.** Correlation matrix showing relatedness of each adiposity variable.

**SI Table 3.** **Bayes Factor (BF).** Showing evidence ratio interpretations.


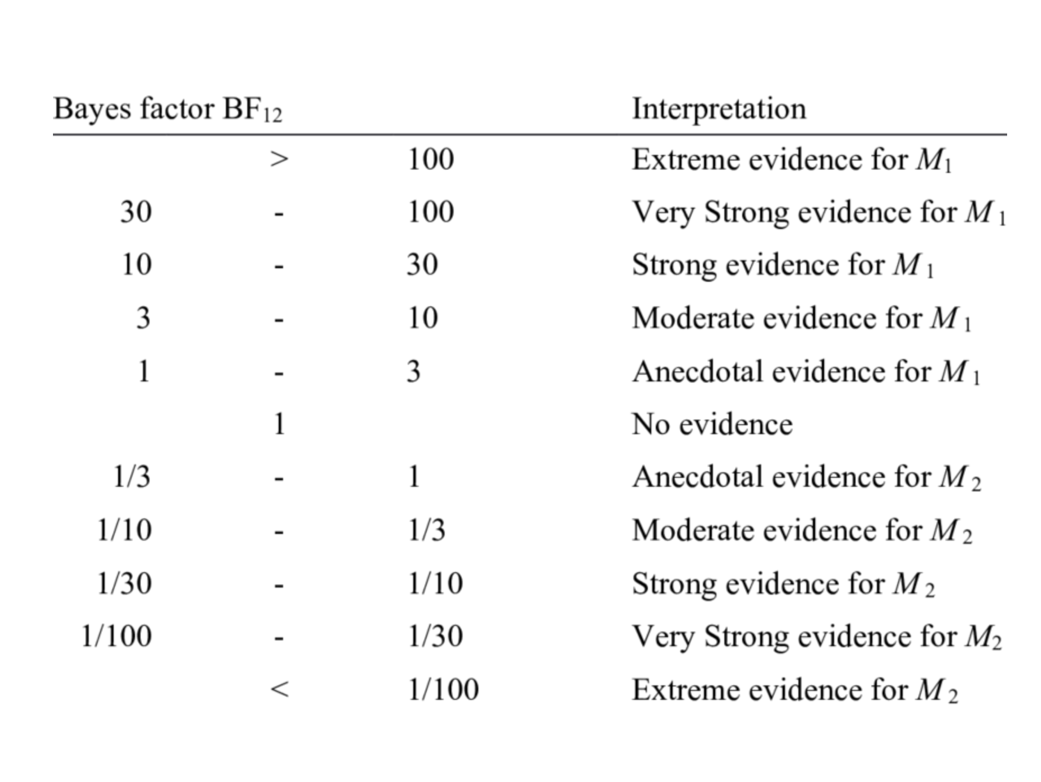


| **SI Table 4.** Average R^2^, root mean square error (RMSE), and mean absolute error (MAE) ± standard deviation for the age prediction models within the training sample (Cam-CAN), test set, and age-corrected test set. | | | | |
| --- | --- | --- | --- | --- |
|  | | Training sample (Cam-CAN) | Test set before age-bias correction | Test set after age-bias correction |
| DTI | R^2^ | 0.82 ± 0.04 | 0.72 | 0.92 |
| RMSE | | 7.67 ± 0.83 | 10.11 | 5.12 |
| MAE | | 6.15 ± 0.55 | 8.37 | 4.06 |
| T1 | R^2^ | 0.81 ± 0.04 | 0.73 | 0.87 |
| RMSE | | 7.93 ± 0.84 | 9.11 | 6.55 |
| MAE | | 6.19 ± 0.83 | 7.2 | 5.21 |

**SI Table 5. Associations between BAGs and adiposity measures.** Showing main effect of each adiposity measure and interaction effects of adiposity measures and time and age.

| modality | cvr | estimate | lower95 | upper95 | p_higher_0 | p_lower_0 | evidence_0 | prior | model |
| --- | --- | --- | --- | --- | --- | --- | --- | --- | --- |
| BAG_DTI | WMR | -0.131 | -0.78 | 0.493 | 0.338 | 0.662 | 2.728 | 1 | Main effect |
| BAG_DTI | Fat_ratio | -0.495 | -1.116 | 0.116 | 0.057 | 0.943 | 0.914 | 1 | Main effect |
| BAG_DTI | Total_Adipose | -0.31 | -0.894 | 0.31 | 0.159 | 0.841 | 2.019 | 1 | Main effect |
| BAG_DTI | VAT_index | -0.048 | -0.658 | 0.557 | 0.437 | 0.563 | 3.196 | 1 | Main effect |
| BAG_DTI | Liver_fat | -0.133 | -0.719 | 0.46 | 0.329 | 0.671 | 2.902 | 1 | Main effect |
| BAG_DTI | WHR | 0.255 | -0.067 | 0.581 | 0.938 | 0.062 | 1.917 | 1 | Main effect |
| BAG_DTI | BMI | -0.211 | -0.519 | 0.081 | 0.084 | 0.916 | 2.619 | 1 | Main effect |
| BAG_DTI | MFI | 0.168 | -0.461 | 0.802 | 0.698 | 0.302 | 2.627 | 1 | Main effect |
| BAG_DTI | TP:WMR | 0.018 | -0.604 | 0.64 | 0.522 | 0.478 | 3.237 | 1 | TP-interaction |
| BAG_DTI | TP:Fat_ratio | -0.144 | -0.683 | 0.41 | 0.304 | 0.696 | 3.117 | 1 | TP-interaction |
| BAG_DTI | TP:Total_Adipose | -0.205 | -0.761 | 0.364 | 0.237 | 0.763 | 2.725 | 1 | TP-interaction |
| BAG_DTI | TP:VAT_index | -0.135 | -0.733 | 0.444 | 0.33 | 0.67 | 2.93 | 1 | TP-interaction |
| BAG_DTI | TP:Liver_fat | -0.213 | -0.914 | 0.434 | 0.267 | 0.733 | 2.513 | 1 | TP-interaction |
| BAG_DTI | TP:WHR | 0.389 | 0.068 | 0.71 | 0.991 | 0.009 | 0.444 | 1 | TP-interaction |
| BAG_DTI | TP:BMI | 0.174 | -0.124 | 0.48 | 0.869 | 0.131 | 3.434 | 1 | TP-interaction |
| BAG_DTI | TP:MFI | 0.43 | -0.279 | 1.135 | 0.88 | 0.12 | 1.406 | 1 | TP-interaction |
| BAG_DTI | Age:WMR | 0.301 | -0.36 | 0.955 | 0.814 | 0.186 | 1.921 | 1 | Age-interaction |
| BAG_DTI | Age:Fat_ratio | 0.176 | -0.44 | 0.769 | 0.716 | 0.284 | 2.75 | 1 | Age-interaction |
| BAG_DTI | Age:Total_Adipose | -0.014 | -0.6 | 0.585 | 0.481 | 0.52 | 3.197 | 1 | Age-interaction |
| BAG_DTI | Age:VAT_index | 0.223 | -0.455 | 0.895 | 0.741 | 0.259 | 2.299 | 1 | Age-interaction |
| BAG_DTI | Age:Liver_fat | 0.173 | -0.559 | 0.898 | 0.678 | 0.322 | 2.26 | 1 | Age-interaction |
| BAG_DTI | Age:WHR | 0.363 | 0.07 | 0.683 | 0.99 | 0.01 | 0.44 | 1 | Age-interaction |
| BAG_DTI | Age:BMI | 0.205 | -0.094 | 0.53 | 0.899 | 0.101 | 2.812 | 1 | Age-interaction |
| BAG_DTI | Age:MFI | 0.486 | -0.187 | 1.156 | 0.919 | 0.081 | 1.064 | 1 | Age-interaction |
| BAG_T1 | WMR | 0.775 | 0.001 | 1.551 | 0.976 | 0.024 | 0.402 | 1 | Main effect |
| BAG_T1 | Fat_ratio | -0.118 | -0.89 | 0.641 | 0.383 | 0.617 | 2.451 | 1 | Main effect |
| BAG_T1 | Total_Adipose | 0.088 | -0.675 | 0.824 | 0.593 | 0.407 | 2.501 | 1 | Main effect |
| BAG_T1 | VAT_index | 0.402 | -0.317 | 1.188 | 0.85 | 0.15 | 1.53 | 1 | Main effect |
| BAG_T1 | Liver_fat | 1.002 | 0.251 | 1.711 | 0.996 | 0.004 | 0.09 | 1 | Main effect |
| BAG_T1 | WHR | 0.173 | -0.232 | 0.602 | 0.794 | 0.206 | 3.4 | 1 | Main effect |
| BAG_T1 | BMI | -0.166 | -0.55 | 0.205 | 0.195 | 0.805 | 3.472 | 1 | Main effect |
| BAG_T1 | MFI | 0.917 | 0.118 | 1.663 | 0.989 | 0.011 | 0.168 | 1 | Main effect |
| BAG_T1 | TP:WMR | 0.675 | -0.076 | 1.402 | 0.962 | 0.038 | 0.569 | 1 | TP-interaction |
| BAG_T1 | TP:Fat_ratio | 0.341 | -0.34 | 1.029 | 0.832 | 0.168 | 1.717 | 1 | TP-interaction |
| BAG_T1 | TP:Total_Adipose | 0.341 | -0.359 | 1.041 | 0.832 | 0.168 | 1.791 | 1 | TP-interaction |
| BAG_T1 | TP:VAT_index | 0.408 | -0.31 | 1.146 | 0.865 | 0.135 | 1.493 | 1 | TP-interaction |
| BAG_T1 | TP:Liver_fat | 0.532 | -0.245 | 1.344 | 0.907 | 0.093 | 1.172 | 1 | TP-interaction |
| BAG_T1 | TP:WHR | 0.5 | 0.104 | 0.919 | 0.991 | 0.009 | 0.302 | 1 | TP-interaction |
| BAG_T1 | TP:BMI | 0.167 | -0.22 | 0.554 | 0.803 | 0.196 | 3.346 | 1 | TP-interaction |
| BAG_T1 | TP:MFI | 0.868 | 0.046 | 1.699 | 0.982 | 0.018 | 0.288 | 1 | TP-interaction |
| BAG_T1 | Age:WMR | 0.999 | 0.201 | 1.818 | 0.992 | 0.008 | 0.131 | 1 | Age-interaction |
| BAG_T1 | Age:Fat_ratio | 0.866 | 0.094 | 1.624 | 0.987 | 0.013 | 0.239 | 1 | Age-interaction |
| BAG_T1 | Age:Total_Adipose | 0.762 | 0.007 | 1.459 | 0.98 | 0.02 | 0.339 | 1 | Age-interaction |
| BAG_T1 | Age:VAT_index | 0.936 | 0.096 | 1.768 | 0.986 | 0.014 | 0.238 | 1 | Age-interaction |
| BAG_T1 | Age:Liver_fat | 0.937 | 0.093 | 1.859 | 0.98 | 0.02 | 0.247 | 1 | Age-interaction |
| BAG_T1 | Age:WHR | 0.683 | 0.29 | 1.076 | 1 | 0 | 0.01 | 1 | Age-interaction |
| BAG_T1 | Age:BMI | 0.42 | 0.025 | 0.822 | 0.98 | 0.02 | 0.618 | 1 | Age-interaction |
| BAG_T1 | Age:MFI | 1.034 | 0.201 | 1.828 | 0.993 | 0.007 | 0.119 | 1 | Age-interaction |


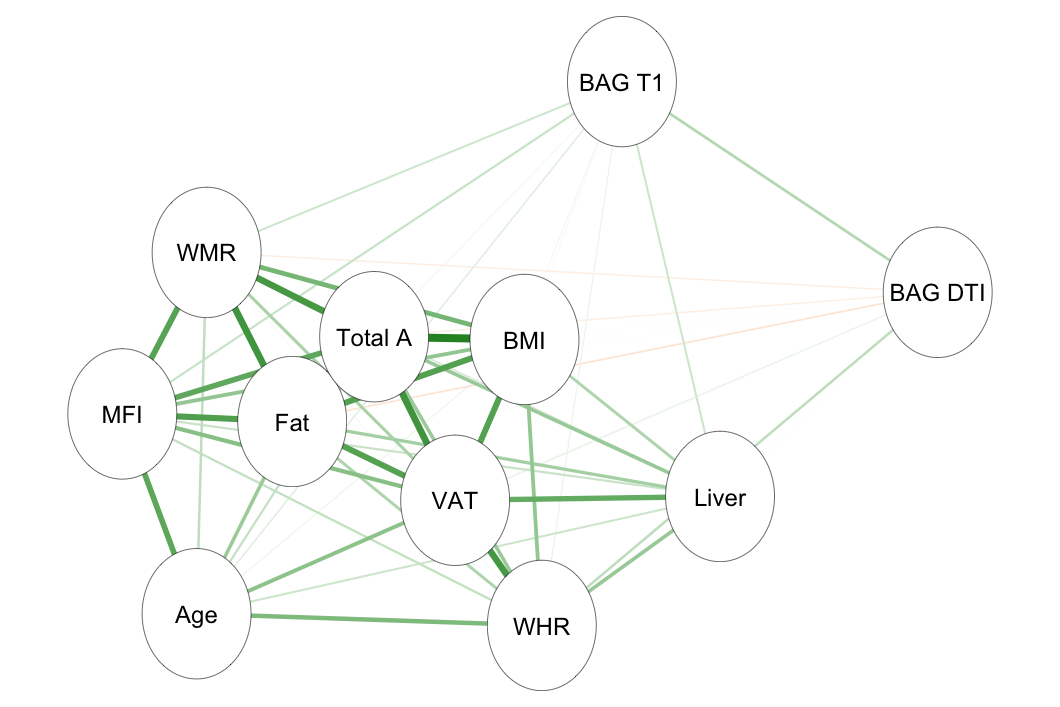


**SI Figure 7.** **Network correlation graph.** Showing correlations between adiposity measures and each BAG. The green lines indicate positive associations, and orange lines (none present) indicate negative associations. Strength of association marked by thickness of each line. Abbreviations: MFI – muscle fat infiltration; Fat – fat ratio; WHR – waist-to-hip ratio; VAT – visceral abdominal tissue index; WMR – weight-to-muscle ratio; Total A – total adipose; BMI – body-mass index; Liver – liver fat.


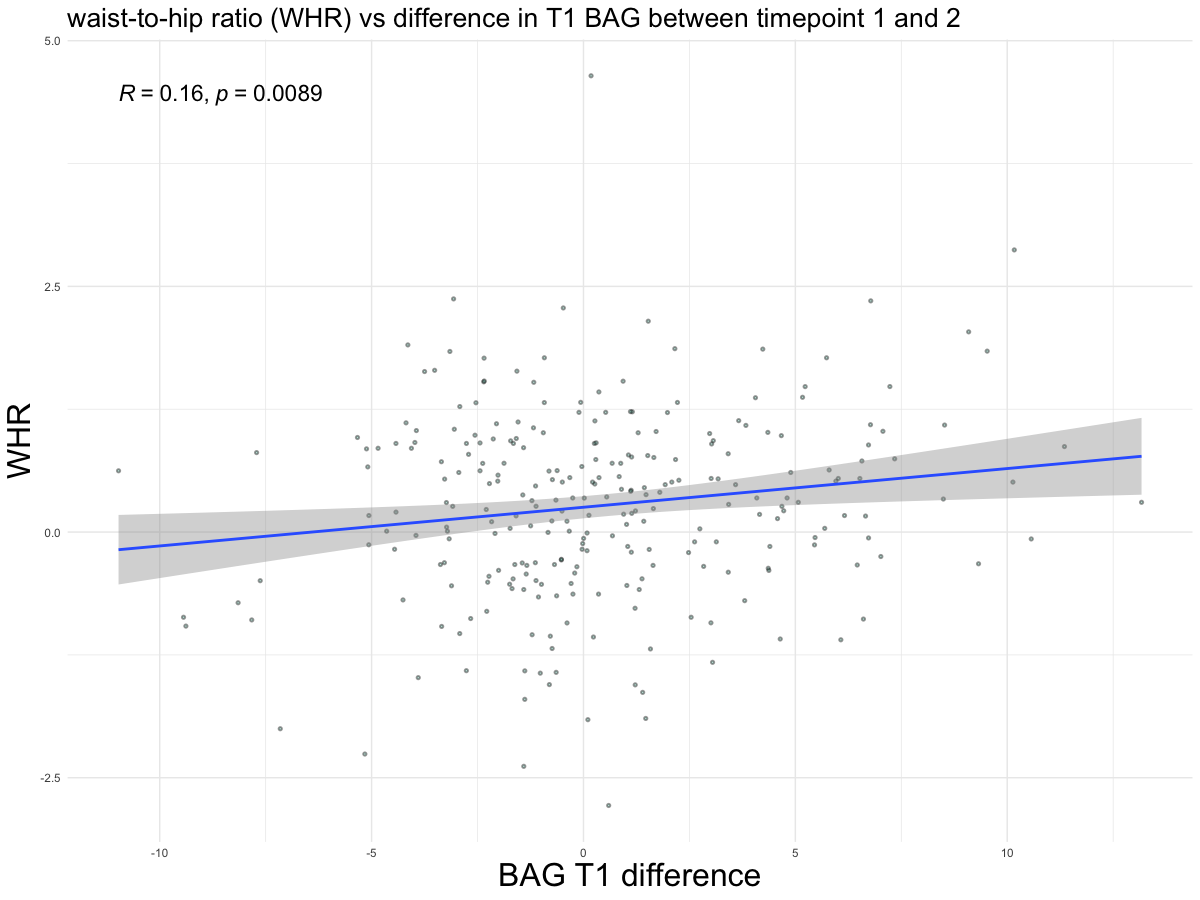


**SI Figure 8. Accelerated brain ageing.** Showing association between wait-to-hip ratio (WHR) and the difference between T1-weighted BAG at timepoint 1 and T1-weighted BAG at timepoint 2. Pearson’s R correlation results and figure show larger differences in T1 BAG delta associated with larger waist-to-hip ratios, indicating accelerated brain ageing.


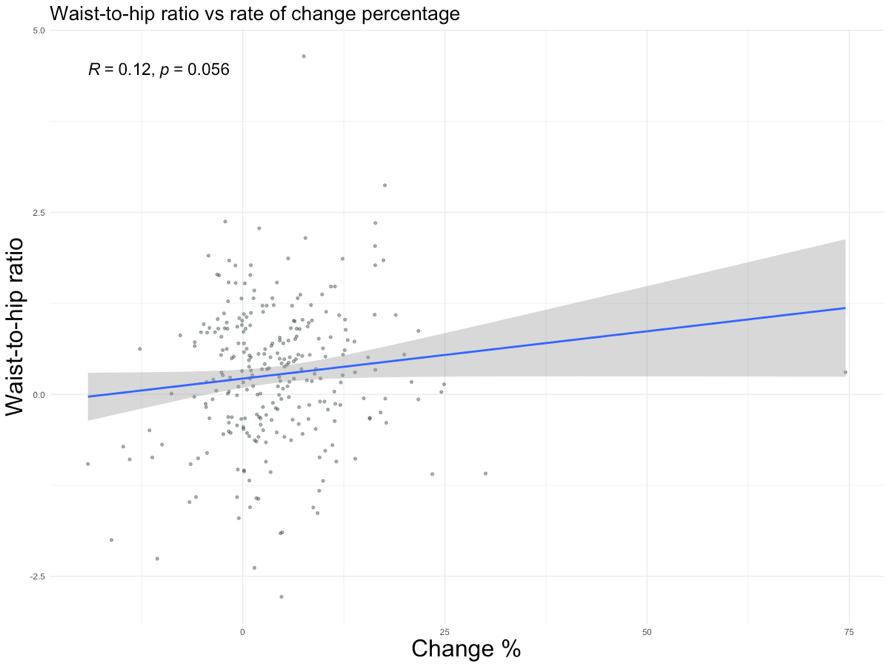


**SI Figure 9.** Waist-to-hip association with rate of change (calculated as a percentage) between T1-weighted brain predicted age at TP1 vs T1-weighted brain predicted age at TP2. Pearson’s R show non-significant results. Note: our longitudinal analyses were carried out with a Bayesian framework with focus on weight of evidence for and against our alternative hypothesis (and null hypothesis) rather than meeting a specific frequentist threshold. An R of 0.12 here likely accurately reflects our longitudinal results of moderate and anecdotal associations.
